# A comparison of Cox and logistic regression for use in genome-wide association studies of cohort and case-cohort design

**DOI:** 10.1038/ejhg.2017.78

**Published:** 2017-05-03

**Authors:** James R Staley, Edmund Jones, Stephen Kaptoge, Adam S Butterworth, Michael J Sweeting, Angela M Wood, Joanna M M Howson

**Affiliations:** 1 Cardiovascular Epidemiology Unit, Department of Public Health and Primary Care, University of Cambridge, Cambridge, UK; 2 The National Institute for Health Research Blood and Transplant Unit (NIHR BTRU) in Donor Health and Genomics at the University of Cambridge, Cambridge, UK

## Abstract

Logistic regression is often used instead of Cox regression to analyse genome-wide association studies (GWAS) of single-nucleotide polymorphisms (SNPs) and disease outcomes with cohort and case-cohort designs, as it is less computationally expensive. Although Cox and logistic regression models have been compared previously in cohort studies, this work does not completely cover the GWAS setting nor extend to the case-cohort study design. Here, we evaluated Cox and logistic regression applied to cohort and case-cohort genetic association studies using simulated data and genetic data from the EPIC-CVD study. In the cohort setting, there was a modest improvement in power to detect SNP–disease associations using Cox regression compared with logistic regression, which increased as the disease incidence increased. In contrast, logistic regression had more power than (Prentice weighted) Cox regression in the case-cohort setting. Logistic regression yielded inflated effect estimates (assuming the hazard ratio is the underlying measure of association) for both study designs, especially for SNPs with greater effect on disease. Given logistic regression is substantially more computationally efficient than Cox regression in both settings, we propose a two-step approach to GWAS in cohort and case-cohort studies. First to analyse all SNPs with logistic regression to identify associated variants below a pre-defined *P*-value threshold, and second to fit Cox regression (appropriately weighted in case-cohort studies) to those identified SNPs to ensure accurate estimation of association with disease.

## Introduction

Cox proportional hazards models are regularly used to analyse time-to-event data in prospective epidemiological cohort and case-cohort studies (Supplementary Text). Case-cohort studies are similar to cohort studies, with the exception that full covariate information is only collected for those individuals who develop the disease over follow-up and a randomly selected subgroup of the initial cohort, referred to as the subcohort (see Supplementary Figure S1). In contrast to traditional epidemiology, logistic regression is often used in genome-wide association studies (GWAS) of cohort and case-cohort data to assess the associations of single-nucleotide polymorphisms (SNPs) and disease outcomes, ignoring the time-to-event information in prospective studies (Supplementary Text).^1, 2^ The reasons for this include the faster computational time of the logistic regression model, the lack of implementation of time-to-event analysis models within most GWAS software and that genetic studies are often combined in multi-study consortia using meta-analysis in which it is convenient to analyse both case–control studies and prospective studies in the same way.

Cox and logistic regression models have been previously compared in cohort studies^3, 4, 5, 6, 7, 8^ but not in case-cohort studies. For cohort studies, it was reported that: (i) the Cox model yields more precise estimates of association; (ii) odds ratios (ORs) and hazard ratios (HRs) diverge as follow-up time, cumulative disease incidence and the strength of the association increase; (iii) in certain situations the Cox model has greater statistical power; and (iv) the Cox model takes a longer time to compute than the logistic regression model. However, most of these studies are at least 20 years old when computational power was limited. Thus, inferences were based on mathematical theory as well as results from small observational studies, and as such do not fully answer the relevant questions for modern GWASs, which are large studies where a small to modest increase in power and precision would probably be sacrificed for an increase in computational efficiency and for practical reasons (such as the availability of suitable software). A more recent study investigated the differences in power between these methods (using simulations) for genetic associations of coronary heart disease (CHD) in a cohort of familial hypercholesterolaemia patients.^9^ The Cox model had significantly greater power compared with logistic regression for SNP–CHD associations in this very high-risk population. However, this study is not generalisable to cohorts with lower disease incidences. Furthermore, practitioners have thus far assumed findings for cohort studies would extrapolate to case-cohort studies, but this has not been formally investigated.

Hence we investigated the potential gains in power and precision against the greater computation time of using Cox proportional hazards models instead of logistic regression models in prospective cohort and case-cohort genetic studies. We performed a simulation study, and validated our findings empirically using SNP–CHD associations from the EPIC-CVD case-cohort study.

## Materials and methods

In the simulation study, we assessed the performance of the Cox and logistic regression models (details of these models are given in the Supplementary Text) in terms of bias, precision and statistical power for cohort and case-cohort studies. The computational time needed to perform a GWAS using both models in cohort and case-cohort studies was also assessed using simulated data. We further compared the Cox and logistic regression models using SNP–CHD associations in EPIC-CVD for loci that have previously been robustly associated with CHD.^1^ All analyses were performed in R (version 3.1.0) on a 24 core Linux server (1.8 GHz, 124Gb RAM).

### Simulation study

We simulated survival data under both the cohort and case-cohort study designs. HR was used as the underlying measure of association and is assumed to be the reference (or gold) standard, and as such, bias was defined as the estimated log(HR) or log(OR) minus the underlying log(HR) (i.e. 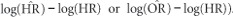. Precision as estimated by the model was assessed using the standard error (SE) of the estimated log(HR) or log(OR). Statistical power was defined (at the per SNP-test level) as the percentage of simulation replications where the SNP–disease association had a *P*-value less than the type I error rate of *α*=0.05.

We considered various combinations of sample size, risk allele frequency (RAF), cumulative disease incidence (proportion of individuals who experienced the disease outcome over the period of follow-up), sampling fraction (for the case-cohort design) and HRs between a single hypothetical SNP and disease, assuming a multiplicative allelic effects model. Sample size was varied over 5000, 10 000 and 25 000 for the cohort study design, and the initial cohort was set to 40 000 for the case-cohort study design. Sampling fractions of 5, 10 and 15% were used in the case-cohort design to generate the subcohort. The additional cases from the initial cohort were then added to create the case-cohort data set. Since the differences between the logistic and Cox model are small for rare diseases,^3, 4, 5^ we selected cumulative disease incidences of 5, 10 and 15% to reflect common diseases (e.g. CHD and type II diabetes) often seen over a 20-year period for the age group studied in the simulations. The RAFs examined were 0.05, 0.10, 0.25, 0.50, 0.75, 0.90 and 0.95. The strength of the association was varied over HRs of 1.05, 1.10, 1.15, 1.20, 1.30, 1.50 and 2.00. To examine the type I error rates of the two approaches, we simulated under an HR of 1. In each simulation scenario the simulation under the null yielded a type I error of approximately 0.05 for both the Cox and logistic regression models (Supplementary Tables S1 and S2).

In each simulation, we constructed a population with an age-structure (in years) similar to that of EPIC-CVD using a normal distribution with mean 56 and standard deviation 6, where all the individuals were assumed to be of the same gender and ethnicity. Genotypes were randomly generated according to the specified RAF using the binomial distribution.

We used the Weibull distribution (with scale parameter λ>0, shape parameter *v*>0 and hazard function *λvt*^*v*−1^) to generate event times^10^ (*T*) assuming a causal effect of age and SNP genotype on disease and proportional hazards,


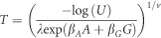


where *T* is survival time (in years); *U* is a random variable following a uniform distribution on [0, 1] *A* is age measured in years; *G* is genotype coded as 0, 1 or 2 according to the number of risk alleles that the individual has; *β*_*A*_ is the log(HR) for a 1 year increase in age; and *β*_*G*_ is the log(HR) for a one allele increase in the risk allele. Approximate estimates of *β*_*A*_ and *λ* for CHD (fatal and non-fatal) were obtained by fitting a Weibull regression model with age as the only covariate in males from the EPIC-CVD subcohort. *β*_*G*_ was set equal to the logarithm of the HR for the hypothetical SNP in each simulation scenario. The shape parameter (*v*) was scaled according to the RAF and underlying HR for the hypothetical SNP to ensure a number of cases that approximately reflects the cumulative disease incidence in each simulation scenario.

We simulated under three censoring distributions with maximum follow-up of 20 years. The first assumed that all the non-cases had complete follow-up of 20 years ('complete follow-up model'). The second assumed each individual was equally likely to be censored at one of four surveys: 5, 10, 15 and 20 years ('survey follow-up model'). The third randomly censored individuals across the 20 years of follow-up using a uniform distribution with minimum 0 years and maximum 20 years ('random follow-up model'). We would expect the censoring distribution of most studies to lie somewhere in-between these distributions. Within each simulation, an individual was considered to have experienced the disease outcome if their event time was less than their censoring time, and to be disease free otherwise. Their time in the study was set to be the minimum of their event time and censoring time.

In the primary simulations, we set the cumulative disease incidence to be either 5, 10 or 15% regardless of the censoring distribution, as model choice is often solely based on the proportion of cases attained over follow-up. However, keeping the proportion of cases constant across censoring strategies changes the disease incidence rate. Hence, we performed a secondary set of simulations where the survey and random censoring strategies were applied to the event time distributions from the complete follow-up model.

We analysed the simulated data using Cox and logistic regression models with genotype and baseline age included as covariates in the model. The Cox model was fitted using the time-on-study time-scale in both the cohort and case-cohort simulations. In the case-cohort setting, Prentice weights and robust SEs were used for the Cox model to account for the sampling process.^11, 12^ Logistic regression in case-cohort studies applied directly to the cases and the subcohort non-cases gives asymptotic inference of odds ratios.^11^ Each simulation scenario was repeated 5000 times. The *P*-values were calculated using Wald tests.

### Computational time

The computational time needed to perform Cox and logistic regression models was estimated using 10 000 variants (with RAFs between 0.05 and 0.95) assuming only one of which is causal with an HR between 1 and 2. In the cohort setting, we used 10 000 simulated individuals. For the case-cohort setting, we generated the case-cohort in the same way described above using a sampling fraction of 15% and an initial cohort of 40 000 simulated individuals. We used the survey follow-up model (see above) and compared the computational times of the models for cumulative disease incidences of 5, 10 and 15%. To estimate these computational times we extended GenABEL^13^ to enable it to perform Prentice-weighted Cox models. This extension is available on request from the authors.

### Empirical example: SNP–CHD associations in EPIC-CVD

EPIC-CVD is a case-cohort study of 31 050 participants derived from 29 recruitment centres across 10 European studies nested within the EPIC study.^14, 15^ The subcohort contains 17 640 individuals, of which 631 had an incident fatal or non-fatal CHD (including angina) event. There were an additional 13 333 CHD events outside the subcohort. The mean age at baseline was 55.3 years and 46.9% of the participants are males. Individuals were genotyped using either the Illumina Cardio-MetaboChip array or the Illumina 660W-Quad BeadChip array. In total, 18 889 individuals had genetic information. We analysed 25 SNPs located within known CHD loci, which were genotyped on both arrays and were available in at least 95% of the genotyped individuals. A multiplicative allelic effects model (on the HR/OR scale) was assumed for the SNPs, and we adjusted for age (in years), sex, EPIC-CVD centre (as a categorical variable) and 10 principal components to adjust for ancestry. Age, sex and EPIC centre were used as covariates to account for differences across EPIC centres (including any differences in the distributions of age and sex). We compared the associations from the Cox and logistic modelling approaches in the full EPIC-CVD case-cohort and in the subcohort of EPIC-CVD (as a surrogate cohort study). The Cox model was fitted using the time-on-study time-scale in both settings. Prentice weights and robust SEs were used for the Cox model in the full case-cohort to account for the sampling process. SNPs were aligned to the plus strand of the human genome reference sequence and are displayed on version 19 (Build 37) of the human genome.

## Results

### Simulation study

#### Cohort study design

**Figure 1:**
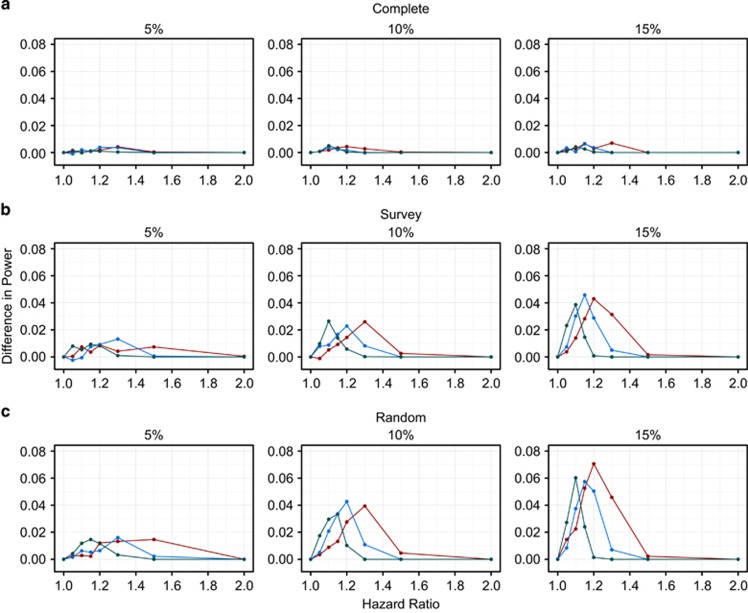
Difference in power between the Cox and logistic regression models for an SNP with a risk allele frequency of 10% for the cohort study design. The red, blue and green lines represent the sample sizes 5000, 10 000 and 25 000, respectively. Complete (**a**), Survey (**b**) and Random (**c**) are the types of follow-up and 5, 10 and 15% are the cumulative disease incidences.

As expected, the power to detect SNP–disease associations increased as the underlying HR increased for both Cox and logistic regression models (Table 1 and Supplementary Table S3). Power also increased as the cumulative disease incidence increased (Table 1 and Supplementary Table S3). The Cox model tended to have more power than logistic regression, and as the cumulative disease incidence increased this difference in power also increased (Figure 1). Furthermore, the increase in power for the Cox model was greater in magnitude as the rate of censoring over the 20 years of follow-up increased (compare Figures 1a–c), from very small differences in power for the complete follow-up model (<1%) to larger differences (up to 7%) for the random follow-up model.

The mean bias for the Cox model was approximately zero across all the simulation scenarios (Table 1 and Supplementary Table S3). However, the estimated ORs were on average larger than the underlying HRs, especially for larger effect sizes. There was also a larger degree of divergence between the HRs and the ORs as the length of follow-up time increased (average follow-up time: complete follow-up model=19.1 years; survey follow-up model=11.9 years; random follow-up model=9.4 years), and as the cumulative disease incidence increased (Table 1 and Supplementary Table S3). The HRs were more precise (as estimated by the model) than the ORs across all scenarios, and this relative difference in precision was positively correlated with cumulative disease incidence (Table 1 and Supplementary Table S3). This increase in precision offsets the greater effect sizes of the logistic regression model, and hence leads to the greater differences in power between the Cox and logistic regression models for the larger cumulative disease incidences (Figure 1).

The differences in power between the Cox and logistic regression models were reasonably similar across the RAFs for each of the combinations of cumulative disease incidence and censoring strategy (Supplementary Figures S2–S4). In the secondary simulations, where the survey and random follow-up censoring strategies were applied to the event time distributions from the complete follow-up model, there was less divergence between the HRs and ORs as the rate of censoring increased (Supplementary Table S4). This decreased divergence, when censoring was introduced, led to a small increase in the greater power of the Cox model for the larger cumulative disease incidences (Supplementary Table S4).

#### Case-cohort study design

**Figure 2:**
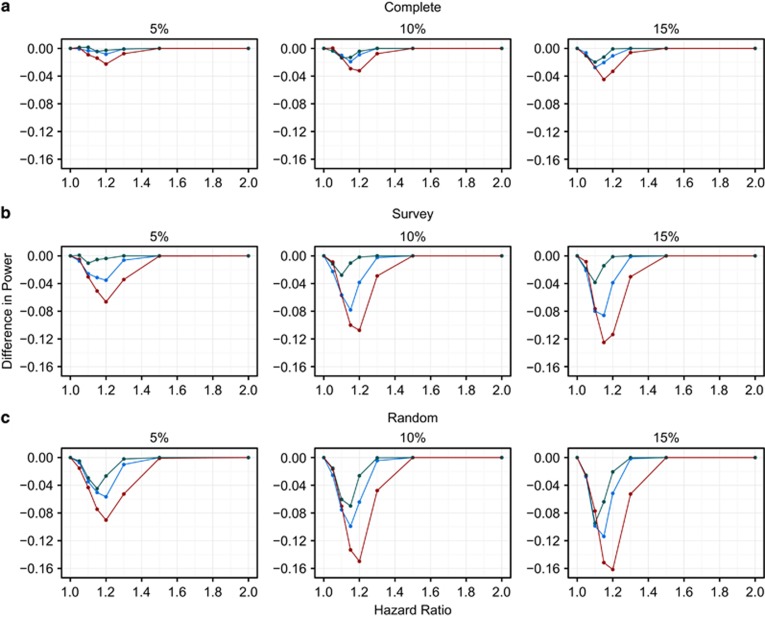
Difference in power between the Cox and logistic regression models for an SNP with a risk allele frequency of 10% for the case-cohort study design. The red, blue and green lines represent the sampling fractions of 5, 10 and 15%, respectively. Complete (**a**), Survey (**b**) and Random (**c**) are the types of follow-up and 5, 10 and 15% are the cumulative disease incidences.

The absolute power increased as the underlying effect size increased for both modelling approaches (Table 2 and Supplementary Table S5). These power curves were steeper as the disease incidence and sampling fraction (which increases the sample size) increased for both models. However, for the case-cohort study design, we found a large decrease in power when using the Prentice-weighted Cox model compared with logistic regression. This loss of power worsened as the cumulative disease incidence increased and as the amount of censoring increased over the 20 years (Figure 2).

The mean bias was approximately zero for the Cox model across all of the scenarios. However, the ORs from the logistic regression model tended to be larger than the underlying HR, and this difference increased as the underlying HR and the length of follow-up increased (Table 2 and Supplementary Table S5). In contrast to the cohort simulations, the HRs were not consistently more precise than the ORs. The mean SEs were smaller for the logistic regression model than the Cox model in the survey and random follow-up models but were slightly larger for the complete follow-up model (Table 2 and Supplementary Table S5). This increased precision of the logistic regression model relative to the Cox model in case-cohort studies (compared with cohort studies) occurs because robust SEs were not required for the logistic regression model but they were for the Cox model to account for the sampling process of this study design. The HRs also became less precise as the average length of follow-up time decreased. For instance, the mean SE of the logarithm of the estimated HR for the sampling fraction of 10%, RAF=0.10, cumulative disease incidence of 10% and underlying HR of 1.10 were 0.052, 0.056 and 0.060 for the complete, survey and random follow-up models, respectively. These large differences in precision between the censoring strategies were caused by the smaller amount of information from the subcohort contributing to the pseudo-likelihood at each failure as the amount of censoring increased. As expected, the distributions of the ORs were similar across the censoring strategies as there were approximately the same number of cases and non-cases across the censoring strategies for the same cumulative disease incidence. Hence, the greater precision of the logistic regression model compared with the Cox model for the random and survey follow-up models leads to the larger differences in power for these models. The increased power of the logistic regression model in the complete follow-up model was mainly driven by the larger effect estimates of the logistic regression model.

The differences in power between the Cox model and the logistic regression models were relatively similar across the RAFs for each of the combinations of cumulative disease incidence and censoring strategy (Supplementary Figures S5–S7). In the secondary simulations, where the event time distributions were kept consistent across the censoring strategies, we observed that there was less divergence between the HRs and ORs as the rate of censoring increased (Supplementary Table S6). There was also a small increase in the difference in power between the two models when there was censoring (Supplementary Table S6). This occurred because, unlike when there was no censoring, the logistic regression model was now more precise than the Cox model (Supplementary Table S6).

### Computational time

The computational time required to complete a genetic association study of 10 000 SNPs using the Cox model was approximately 18 times greater than the equivalent analysis using logistic regression for cohort studies (cohort size of 10 000), regardless of the cumulative disease incidence (Supplementary Table S7). Similarly, the computational time needed to analyse 10 000 SNPs with the Cox model was at least 190 times greater than the same analysis using logistic regression for case-cohort studies (sampling fraction of 15% initial cohort of 40 000). We expect these relative differences in computational time between the models to remain the same for data sets with greater numbers of SNPs.

### Empirical example: SNP–CHD associations in EPIC-CVD

To assess Cox and logistic regression models in cohort studies, we used the subcohort of EPIC-CVD and 25 SNPs from known CHD loci. Since there were only 437 CHD events in the individuals genotyped in the subcohort, there was limited power to detect SNP–CHD associations in the subcohort with either model. Nevertheless, in the subcohort the effect estimates were directionally concordant for the Cox and logistic regression models for 24 of the 25 SNPs (Table 3), and the SNP that was directionally discordant had effect estimates very close to the null for both models. Of the 24 concordant SNPs, 18 had the same direction of effect as in the literature (Supplementary Table S8).^1, 2^ The SNPs that showed different directions of association with the literature were in general those SNPs with smaller effect on CHD and hence required large sample sizes to detect these effects (Supplementary Table S8). The Cox model was more precise than logistic regression for all of the SNPs and for the effect sizes further away from the null the ORs tended to be larger in magnitude (Table 3).

We assessed Cox and logistic regression models in case-cohort studies by using the entire EPIC-CVD case-cohort and the same 25 SNPs as above. Here, the effect estimates were directionally concordant for both models for 22 of the 25 SNPs (Table 3), of which 21 had the same direction of effect as in the literature (Supplementary Table S8). Like in the subcohort, the effect estimates of the SNPs that were directionally discordant between the models lie close to the null and the SNP that was directionally discordant with the literature had a small effect on CHD in the large consortia (Supplementary Table S8). The logistic regression model was more precise than the Cox model for all of the SNPs often leading to much smaller *P*-values. For instance, the SNP rs974819 (chr11:g.103660567C>T; an SNP located downstream of *PDGFD*) had similar effect sizes for the Cox (log(HR)=−0.082) and logistic (log(OR)=−0.080) regression models, but the SEs were 0.031 and 0.027, respectively. This led to a smaller *P*-value for the logistic regression model (*P*=0.003 compared with *P*=0.007). Again, the effect sizes of the logistic regression model tended to be larger in magnitude than those of the Cox model for the effect estimates of greater magnitude. Two examples of this are the SNPs rs9349379 (chr6:g.12903957A>G; an intronic variant in *PHACTR1*) and rs2075650 (chr19:g.45395619A>G; an intronic variant in *TOMM40* upstream of *APOE*) where the effect sizes were 0.03 and 0.013 larger in magnitude for the logistic regression model compared with the Cox model. This in combination with smaller SEs for the logistic regression model led to much smaller *P*-values for this model (Table 3).

## Discussion

In this paper, we have investigated the differences between the Cox and logistic regression models in cohort and case-cohort studies using simulations and SNP–CHD associations from EPIC-CVD. In the simulations, we examined a wide range of scenarios, including varying the amount of censoring (over a 20-year follow-up period), the RAF of the SNP, the strength of the association and the number of events (by changing the cumulative disease incidence). The results from the EPIC-CVD case-cohort show similar findings to the simulations. We believe that this is the first study to specifically compare the Cox and logistic regression models in case-cohort studies.

We have shown that there is increased power to detect SNP–disease associations using the Cox model instead of logistic regression for the cohort study design and the extent to which it has additional power depends on cumulative disease incidence and the length of follow-up. However, the increase in power using the Cox model was small when the cumulative disease incidence was low or when the non-cases had complete follow-up. We also observed that the ORs and HRs diverge as follow-up time and cumulative disease incidence increase (especially for large underlying effects), that the HRs were more precise, and logistic regression was more computationally efficient. These findings are in line with and extend those from previous studies with the emphasis in the current work being the application of these modelling approaches in genetic association studies.^4, 5, 7, 8, 9^

In contrast to the cohort study design, it appears that there is no additional power to be gained by using Prentice-weighted Cox models for genetic associations in case-cohort studies. Indeed, there is a striking loss in power, which occurs because robust SEs are not necessary for the logistic regression model in addition to its effect estimates being greater in magintude. Furthermore, the computational cost of the Cox model was far greater than that of the logistic regression model. Hence, although we recognise the caveats of using the logistic regression model (inflated effect estimates, especially for associations of greater magnitude) in case-cohort studies, we propose that logistic regression could be used as a filter to detect SNPs below a pre-defined *P*-value threshold for GWAS (of a large number of SNPs (>500 000)) in case-cohort studies. This threshold should be set suitably high to ensure that all of the SNPs that would have been detected at the overall level of significance with the Cox model are contained within this subset, for example, 1 × 10^−4^ if the overall significance threshold is 5 × 10^−8^. Prentice-weighted Cox models could then be fitted to this subset of SNPs, avoiding the vast computational time required to complete an entire GWAS using the Prentice-weighted Cox model, while obtaining accurate estimates of effect and inference (including *P*-values) for the SNPs of particular interest. This two-step procedure could also be applied in the cohort setting to reduce computational time.

We must note here that HRs and ORs are different measures of association and have different interpretations. Cox models incorporate the length of time the individuals are followed up and measure whether the risk factor affects the time at which the disease event occurs. Logistic regression assesses whether the risk factor affects the odds of disease, and hence does not take into account the time of disease occurrence. So early and late failures are given the same weight in the analysis. In addition, individuals who are not observed to have the event during the period of follow-up time are treated as controls. This is different from the time-to-event analysis approaches where these individuals are considered to be censored. That is, we assume that all the individuals will have the event at some point but we just do not observe this event over the follow-up period for some individuals. Since these models have different definitions and estimate different parameters, naturally the results from these models will differ, as observed in our work.

The results of our simulations and EPIC-CVD analyses are directly generalisable to diseases with cumulative incidence between 5 and 15% over a 20-year period of follow-up. However, our results could probably be safely extrapolated to other disease incidences over this period of follow-up (i.e. the Cox and logistic model estimates will become increasingly similar as the disease becomes rarer (cumulative disease incidence<5%) and will diverge more as the disease becomes more common (cumulative disease incidence>15%)). Additional avenues of research could include comparing further time-to-event models (e.g. parametric survival models), examining the effect of violating the proportional hazards assumption, meta-analysing case–control studies alongside prospective studies and the inclusion of prevalent cases in the analysis.

In summary, to minimise computational time in analysing genetic associations in cohort and case-cohort studies, while obtaining appropriate estimates of effect for SNPs of greater interest we propose a two-step procedure. Firstly, logistic regression is used to analyse all SNPs as an initial filtering process and secondly, Cox regression is fitted to those SNPs associated below a pre-defined *P*-value threshold to avoid inflated estimates of effect as well as to obtain appropriate *P*-values for these SNPs. However, logistic regression remains a practical alternative to Cox models in cohort and case-cohort studies, especially when the disease incidence is low.

## Figures and Tables

**Table 1:** Simulation results for cohort studies with 10,000 individuals for a SNP with RAF=0.10.

	*Cox Regression (HR)*			*Logistic Regression (OR)*			
*True HR*	*Mean(Bias)*	*Mean(SE)*	*Power*	*Mean(Bias)*	*Mean(SE)*	*Power*	*Diff in Power*
Complete Follow-up, 5% Cumulative Disease Incidence							
1.1	–0.0039	0.1020	0.1610	–0.0010	0.1052	0.1590	0.0020
1.2	–0.0024	0.0985	0.4674	0.0034	0.1018	0.4636	0.0008
1.5	–0.0031	0.0909	0.9856	0.0111	0.0947	0.9858	–0.0002
2	–0.0014	0.0834	1.0000	0.0264	0.0881	1.0000	0.0000
Complete Follow-up, 10% Cumulative Disease Incidence							
1.1	–0.0025	0.0716	0.2664	0.0036	0.0764	0.2628	0.0036
1.2	–0.0006	0.0694	0.7360	0.0114	0.0744	0.7342	0.0022
1.5	–0.0004	0.0642	0.9998	0.0290	0.0699	0.9998	0.0000
2	–0.0005	0.0586	1.0000	0.0576	0.0655	1.0000	0.0000
Complete Follow-up, 15% Cumulative Disease Incidence							
1.1	–0.0008	0.0588	0.3754	0.0087	0.0648	0.3748	0.0006
1.2	–0.0013	0.0570	0.8730	0.0168	0.0633	0.8694	0.0036
1.5	–0.0007	0.0530	1.0000	0.0440	0.0601	1.0000	0.0000
2	–0.0007	0.0487	1.0000	0.0869	0.0574	1.0000	0.0000
							
Survey Follow-up, 5% Cumulative Disease Incidence							
1.1	–0.0006	0.1011	0.1678	0.0020	0.1044	0.1684	-0.0006
1.2	–0.0015	0.0979	0.4668	0.0026	0.1013	0.4576	0.0092
1.5	–0.0043	0.0907	0.9864	0.0065	0.0945	0.9858	0.0006
2	–0.0014	0.0840	1.0000	0.0193	0.0885	1.0000	0.0000
Survey Follow-up, 10% Cumulative Disease Incidence							
1.1	–0.0035	0.0716	0.2638	–0.0006	0.0763	0.2550	0.0088
1.2	–0.0016	0.0694	0.7296	0.0057	0.0743	0.7068	0.0228
1.5	–0.0019	0.0645	1.0000	0.0166	0.0700	1.0000	0.0000
2	0.0002	0.0594	1.0000	0.0362	0.0661	1.0000	0.0000
Survey Follow-up, 15% Cumulative Disease Incidence							
1.1	–0.0018	0.0590	0.3672	0.0027	0.0650	0.3370	0.0302
1.2	–0.0005	0.0573	0.8760	0.0093	0.0635	0.8472	0.0288
1.5	–0.0011	0.0535	1.0000	0.0219	0.0605	1.0000	0.0000
2	–0.0013	0.0498	1.0000	0.0439	0.0580	1.0000	0.0000
							
Random Follow-up, 5% Cumulative Disease Incidence							
1.1	–0.0038	0.1017	0.1682	–0.0022	0.1049	0.1620	0.0062
1.2	–0.0025	0.0983	0.4638	0.0004	0.1016	0.4574	0.0064
1.5	–0.0031	0.0913	0.9858	0.0049	0.0950	0.9836	0.0022
2	–0.0037	0.0852	1.0000	0.0106	0.0896	1.0000	0.0000
Random Follow-up, 10% Cumulative Disease Incidence							
1.1	–0.0018	0.0721	0.2702	0.0005	0.0768	0.2494	0.0208
1.2	–0.0025	0.0699	0.7324	0.0017	0.0748	0.6898	0.0426
1.5	–0.0029	0.0651	1.0000	0.0078	0.0706	1.0000	0.0000
2	–0.0006	0.0604	1.0000	0.0198	0.0668	1.0000	0.0000
Random Follow-up, 15% Cumulative Disease Incidence							
1.1	–0.0016	0.0589	0.3614	–0.0003	0.0648	0.3240	0.0374
1.2	–0.0012	0.0572	0.8786	0.0031	0.0634	0.8282	0.0504
1.5	–0.0011	0.0536	1.0000	0.0091	0.0605	1.0000	0.0000
2	–0.0008	0.0502	1.0000	0.0184	0.0582	1.0000	0.0000

Abbreviations: Diff in Power, difference in power (Cox − logistic); HR, hazard ratio; OR, odds ratio; RAF, risk allele frequency; SE, standard error of the logarithm of hazard or odds ratio. Bias refers to either the estimated log(HR) or log(OR) minus the underlying log(HR). It is important to note that HRs and ORs are different measures of comparison and as such ORs are not ’biased‘ if different from the underlying HR. This table is a subset of Supplementary Table S3.

**Table 2:** Simulation results for case-cohort studies with sampling fraction of 10% from 40,000 individuals for a SNP with RAF=0.10.

	*Cox Regression (HR)*			*Logistic Regression (OR)*			
*True HR*	*Mean(Bias)*	*Mean(SE)*	*Power*	*Mean(Bias)*	*Mean(SE)*	*Power*	*Diff in Power*
Complete Follow-up, 5% Cumulative Disease Incidence							
1.1	–0.0007	0.0635	0.3284	0.0021	0.0647	0.3318	–0.0034
1.2	–0.0002	0.0622	0.8308	0.0053	0.0634	0.8392	–0.0084
1.5	0.0010	0.0596	1.0000	0.0143	0.0608	1.0000	0.0000
2	0.0007	0.0582	1.0000	0.0259	0.0588	1.0000	0.0000
Complete Follow-up, 10% Cumulative Disease Incidence							
1.1	0.0001	0.0524	0.4480	0.0054	0.0542	0.4580	–0.0100
1.2	0.0006	0.0517	0.9430	0.0119	0.0536	0.9522	–0.0092
1.5	–0.0001	0.0505	1.0000	0.0270	0.0524	1.0000	0.0000
2	0.0004	0.0503	1.0000	0.0531	0.0518	1.0000	0.0000
Complete Follow-up, 15% Cumulative Disease Incidence							
1.1	0.0011	0.0484	0.5126	0.0098	0.0510	0.5402	–0.0276
1.2	–0.0008	0.0481	0.9674	0.0161	0.0507	0.9782	–0.0108
1.5	0.0003	0.0475	1.0000	0.0418	0.0504	1.0000	0.0000
2	0.0022	0.0479	1.0000	0.0816	0.0507	1.0000	0.0000
							
Survey Follow-up, 5% Cumulative Disease Incidence							
1.1	0.0002	0.0659	0.3120	0.0027	0.0643	0.3378	–0.0258
1.2	0.0008	0.0647	0.8050	0.0049	0.0631	0.8402	–0.0352
1.5	–0.0002	0.0624	1.0000	0.0094	0.0607	1.0000	0.0000
2	0.0015	0.0612	1.0000	0.0195	0.0590	1.0000	0.0000
Survey Follow-up, 10% Cumulative Disease Incidence							
1.1	–0.0004	0.0569	0.3786	0.0026	0.0541	0.4350	–0.0564
1.2	0.0009	0.0563	0.9038	0.0076	0.0536	0.9422	–0.0384
1.5	0.0007	0.0553	1.0000	0.0171	0.0525	1.0000	0.0000
2	0.0024	0.0551	1.0000	0.0334	0.0519	1.0000	0.0000
Survey Follow-up, 15% Cumulative Disease Incidence							
1.1	0.0019	0.0542	0.4332	0.0064	0.0510	0.5130	–0.0798
1.2	–0.0005	0.0538	0.9294	0.0089	0.0507	0.9682	–0.0388
1.5	0.0016	0.0533	1.0000	0.0230	0.0504	1.0000	0.0000
2	0.0008	0.0535	1.0000	0.0424	0.0506	1.0000	0.0000
							
Random Follow-up, 5% Cumulative Disease Incidence							
1.1	–0.0007	0.0681	0.2864	–0.0001	0.0645	0.3212	–0.0348
1.2	0.0005	0.0670	0.7768	0.0030	0.0633	0.8334	–0.0566
1.5	–0.0005	0.0648	1.0000	0.0067	0.0609	1.0000	0.0000
2	0.0007	0.0639	1.0000	0.0143	0.0593	1.0000	0.0000
Random Follow-up, 10% Cumulative Disease Incidence							
1.1	0.0006	0.0599	0.3536	0.0021	0.0543	0.4290	–0.0754
1.2	–0.0005	0.0593	0.8756	0.0037	0.0537	0.9398	–0.0642
1.5	0.0004	0.0583	1.0000	0.0097	0.0526	1.0000	0.0000
2	0.0004	0.0583	1.0000	0.0192	0.0520	1.0000	0.0000
Random Follow-up, 15% Cumulative Disease Incidence							
1.1	–0.0002	0.0574	0.3740	0.0016	0.0509	0.4726	–0.0986
1.2	0.0021	0.0571	0.9062	0.0055	0.0507	0.9578	–0.0516
1.5	0.0004	0.0566	1.0000	0.0112	0.0503	1.0000	0.0000
2	0.0031	0.0571	1.0000	0.0199	0.0505	1.0000	0.0000

Abbreviations: Diff in Power, difference in power (Cox − logistic); HR, hazard ratio; OR, odds ratio; RAF, risk allele frequency; SE, standard error of the logarithm of hazard or odds ratio. Bias refers to either the estimated log(HR) or log(OR) minus the underlying log(HR). It is important to note that HRs and ORs are different measures of comparison and as such ORs are not 'biased' if different from the underlying HR. The Cox model was Prentice weighted and robust SEs were applied to account for the sampling process. This table is a subset of Supplementary Table S5.

**Table 3:** Results of 25 SNPs previously reported to be associated with CHD in EPIC-CVD

		*Subcohort*							*Case-cohort*						
			*Cox regression*			*Logistic regression*				*Cox regression*			*Logistic regression*		
*rsID*	*Build 37 chr:pos*	N (N *cases*)	*β*	*SE*	P	*β*	*SE*	P	N (N *cases*)	*β*	*SE*	P	*β*	*SE*	P
rs11206510	chr1:g.55496039T>C	11 812 (437)	0.058	0.088	0.509	0.052	0.092	0.572	18 807 (7432)	0.069	0.036	0.054	0.073	0.031	0.021
rs4299376	chr2:g.44072576T>G	11 697 (436)	−0.020	0.073	0.785	−0.011	0.077	0.885	18 653 (7392)	−0.058	0.030	0.053	−0.043	0.026	0.098
rs2252641	chr2:g.145801461T>C	11 799 (437)	−0.130	0.068	0.057	−0.138	0.072	0.054	18 785 (7423)	−0.019	0.028	0.508	−0.012	0.025	0.639
rs2306374	chr3:g.138119952T>C	11 812 (437)	−0.116	0.090	0.199	−0.125	0.095	0.189	18 806 (7431)	0.008	0.038	0.843	−0.008	0.033	0.821
rs273909	chr5:g.131667353A>G	11 789 (434)	0.012	0.107	0.909	−0.001	0.112	0.996	18 757 (7402)	−0.049	0.043	0.260	−0.065	0.038	0.083
rs2706399	chr5:g.131867702A>G	11 812 (437)	−0.040	0.068	0.552	−0.057	0.071	0.428	18 807 (7431)	0.028	0.028	0.319	−0.015	0.025	0.556
rs9349379	chr6:g.12903957A>G	11 811 (437)	−0.127	0.069	0.066	−0.133	0.073	0.069	18 806 (7432)	−0.093	0.029	0.001	−0.123	0.025	9.00E-07
rs2048327	chr6:g.160863532T>C	11 813 (437)	0.042	0.071	0.553	0.042	0.075	0.578	18 808 (7432)	−0.083	0.030	0.005	−0.092	0.026	4.00E-04
rs2023938	chr7:g.19036775T>C	11 809 (437)	0.000	0.112	1.000	0.001	0.118	0.992	18 802 (7430)	−0.018	0.046	0.691	−0.043	0.040	0.286
rs10953541	chr7:g.107244545C>T	11 810 (437)	0.061	0.081	0.450	0.065	0.084	0.438	18 805 (7432)	−0.009	0.033	0.792	0.015	0.029	0.607
rs264	chr8:g.19813180G>A	11 812 (437)	0.140	0.098	0.151	0.141	0.102	0.168	18 807 (7432)	0.079	0.039	0.040	0.076	0.035	0.029
rs10808546	chr8:g.126495818C>T	11 787 (434)	0.033	0.070	0.632	0.034	0.073	0.642	18 769 (7416)	0.042	0.028	0.142	0.036	0.025	0.149
rs3217992	chr9:g.22003223C>T	11 813 (437)	−0.034	0.070	0.633	−0.036	0.074	0.629	18 808 (7432)	−0.114	0.029	8.00E-05	−0.097	0.025	1.00E-04
rs2047009	chr10:g.44539913G>T	11 811 (437)	0.104	0.068	0.130	0.106	0.072	0.140	18 797 (7432)	0.055	0.028	0.049	0.072	0.024	0.003
rs1746048	chr10:g.44775824C>T	11 811 (437)	0.101	0.104	0.329	0.110	0.108	0.309	18 805 (7432)	0.060	0.042	0.146	0.062	0.036	0.082
rs11203042	chr10:g.90989109C>T	11 813 (437)	0.034	0.069	0.618	0.037	0.072	0.610	18 808 (7432)	0.014	0.028	0.612	0.005	0.025	0.829
rs1412444	chr10:g.91002927C>T	11 812 (437)	−0.118	0.071	0.097	−0.128	0.075	0.086	18 806 (7431)	−0.001	0.030	0.962	−0.041	0.026	0.111
rs974819	chr11:g.103660567C>T	11 324 (424)	−0.123	0.073	0.091	−0.132	0.077	0.086	18 163 (7263)	−0.082	0.031	0.007	−0.080	0.027	0.003
rs964184	chr11:g.116648917C>G	11 811 (437)	0.126	0.103	0.221	0.128	0.108	0.236	18 806 (7432)	−0.047	0.040	0.241	−0.063	0.034	0.065
rs3184504	chr12:g.111884608C>T	11 800 (437)	0.049	0.068	0.472	0.054	0.071	0.447	18 755 (7392)	−0.003	0.028	0.919	−0.014	0.025	0.560
rs9319428	chr13:g.28973621G>A	11 808 (437)	−0.158	0.072	0.027	−0.165	0.075	0.028	18 800 (7429)	−0.050	0.030	0.098	−0.057	0.026	0.030
rs4380028	chr15:g.79111093C>T	11 812 (437)	0.082	0.070	0.241	0.088	0.073	0.229	18 807 (7432)	0.086	0.029	0.003	0.065	0.025	0.010
rs17514846	chr15:g.91416550C>A	11 811 (437)	−0.115	0.068	0.091	−0.112	0.072	0.119	18 796 (7422)	−0.037	0.028	0.187	−0.068	0.025	0.006
rs6511720	chr19:g.11202306G>T	11 813 (437)	−0.067	0.105	0.525	−0.063	0.110	0.568	18 807 (7432)	0.062	0.044	0.156	0.067	0.039	0.089
rs2075650	chr19:g.45395619A>G	11 813 (437)	−0.093	0.099	0.347	−0.099	0.104	0.340	18 808 (7432)	−0.120	0.041	0.003	−0.133	0.036	2.00E-04

*N*, number of individuals; Build 37 chr:pos, dbSNP (hg19) chromosome:position (risk allele>other allele); *β* is the logarithm of the association measure (either odds ratio or hazard ratio); SE, standard errors of the logarithm of the association measure (either odds ratio or hazard ratio). In the full case-cohort, the Cox model was Prentice weighted. Alleles are aligned to the + strand.
