# Supplementary material for: A comparison of Cox and logistic regression for use in genome-wide association studies of cohort and case-cohort design
Source: Eur J Hum Genet. 2017 May 3;25(7):854–62. doi: 10.1038/ejhg.2017.78 (PMC5520083; doi:10.1038/ejhg.2017.78)
Supplement: Supplementary Information [file ejhg201778x1.pdf]

A comparison of Cox and logistic regression for use in genome-wide association studies of cohort and case-cohort design: Supplementary Data

James R Staley[1], Edmund Jones[1], Stephen Kaptoge[1], Adam S Butterworth[1,2], Michael Sweeting[1,2], Angela M Wood[1,2], Joanna M M Howson[1], on behalf of the EPIC-CVD Consortium.

[1]Cardiovascular Epidemiology Unit, Department of Public Health and Primary Care, University of Cambridge, United Kingdom.

[2]The National Institute for Health Research Blood and Transplant Unit (NIHR BTRU) in Donor Health and Genomics at the University of Cambridge, United Kingdom.

Correspondence:

Mr James Staley or Dr Joanna Howson

Department of Public Health & Primary Care

Strangeways Research Laboratory

Wort's Causeway

Cambridge

CB1 8RN

UK

Email: jrs95@medschl.cam.ac.uk or jmmh2@medschl.cam.ac.uk

Telephone: +44 (0) 1223 748655

EPIC-CVD Investigators

Aarhus: Kim Overvad[1]; **Asturias:** J. Ramón Quirós[2]; **Athens:** Antonia Trichopoulou[3], Michalis Katsoulis[3], Nikos Yiannakouris[3]; **Bilthoven:** Jolanda MA Boer[4], W.M.Monique Verschuren[4,5]; **Copenhagen:** Anne Tjønneland[6]; **Florence:** Domenico Palli; **Granada:** Emilio Sánchez-Cantalejo; **Heidelberg:** Rudolf Kaaks[7], Myrto Barrdahl[7]; **Imperial:** Camille Lassale[8], Ioanna Tzoulaki[8], Elio Riboli[8]; **Malmö:** Olle Melander[9]; **Milan:** Vittorio Krogh[10], Claudia Agnoli[10]; **Murcia:** Diego Salmerón[11,12,13]; **Naples:** Amalia Mattiello[14]; **Navarra:** Conchi Moreno-Iribas[15]; **Norfolk:** Nicholas J Wareham[16], Claudia Langenberg[16], Robert Scott[16], Stephen Sharp[16], Nita G Forouhi[16]; **Paris:** Laura Baglietto[17,18]; **Potsdam:** Heiner Boeing[19]; **Ragusa:** Rosario Tumino[20]; **San Sebastian:** Larraitz Arriola[21]; **Tromsø:** Elisabete Weiderpass[22,23,24,25], Eiliv Lund[22,23,24,25]; **Turin:** Carlotta Sacerdote[26]; **Umea:** Jan-Håkan Jansson[27,28,29]; Frida Renström[27,28,29]; **Utrecht:** Yvonne van der Schouw[30], Carl Moons[30].

[1]Aarhus University, Department of Public Health, Section for Epidemiology, Bartholins Alle 2, DK-8000 Aarhus C, Denmark; [2]Public Health Directorate, Asturias, Spain; [3]Hellenic Health Foundation, Athens, Greece; [4]Centre for Nutrition, Prevention and Health Services, National Institute for Public Health and the Environment (RIVM), Bilthoven, the Netherlands; [5]Julius Center for Health Sciences and Primary Care, University Medical Center Utrecht, Utrecht, the Netherlands; [6]Diet, Genes and Environment, Danish Cancer Society Research Center, Strandboulevarden 49, DK 2100 Copenhagen ø Denmark; [7]Division of Cancer Epidemiology, German Cancer Research Center (DKFZ) im Neuenheimer Feld 581, 69121 Heidelberg, Germany; [8]Department of Epidemiology and Biostatistics, Imperial College London; [9]CRR, Lund University, 20502 Malmö; [10]Epidemiology and Prevention Unit, Fondazione IRCCS Istituto Nazionale dei Tumori - Milan; [11]Department of Epidemiology, Murcia Regional Health Council, IMIB-Arrixaca, Murcia, Spain; [12]CIBER Epidemiología y Salud Pública (CIBERESP), Spain; [13]Department of Health and Social Sciences, Universi-

dad de Murcia, Spain; [14]Dipartimento di Medicina Clinica e Chirurgia, Federico II University, Naples, Italy; [15]Moreno-Iribas C, Instituto de Salud Pública de Navarra, IdiSNA - Navarra Institute for Health Research; [16]MRC Epidemiology Unit, University of Cambridge; [17]Cancer Epidemiology Centre, Cancer Council of Victoria, Melbourne, Victoria, Australia; [18]Centre for Molecular, Environmental, Genetic and Analytic Epidemiology, School of Population Health, University of Melbourne, Victoria, Australia; [19]Department of Epidemiology, German Institute of Human Nutrition Potsdam-Rehbruecke; [20]Cancer Registry and Histopathology Unit, "Civic - M.P.Arezzo" Hospital, ASP Ragusa (Italy); [21]Public Health Division of Gipuzkoa, Instituto BIO-Donostia, Basque Government, Instituto BIO-Donostia, Basque Government CIBER Epidemiología y Salud Pública - CIBERESP, Spain; [22]Department of Community Medicine, University of Tromsø, The Arctic University of Norway, Tromsø, Norway; [23]Department of Research, Cancer Registry of Norway, Institute of Population Based Cancer Research, Oslo, Norway; [24]Department of Medical Epidemiology and Biostatistics, Karolinska Institute, Stockholm, Sweden; [25]Genetic Epidemiology Group, Folkhälsan Research Center, Helsinki, Finland; [26]Unit of Cancer Epidemiology, Piedmont Children Cancer Registry, Città della Salute e della Scienza di Torino Hospital, Turin, Italy; [27]Department of Public Health and Clinical Medicine, Skellefteå Research Unit, Umeå University, Sweden; [28]Genetic & Molecular Epidemiology Unit, Department of Clinical Sciences, Lund University, Malmö, Sweden; [29]Department of Biobank Research, Umeå University, Umeå, Sweden; [30]Julius Center for Health Sciences and Primary Care, University Medical Center Utrecht, Utrecht, The Netherlands.

Supplementary Text

Logistic regression

Logistic regression models[1,2] are often used to analyse binary data (e.g. disease status), and can be described in the generalised linear model framework. Suppose that y_i is the realisation of a random variable Y_i for the i-th individual, where $Y_i = 1$ if the individual has the disease and 0 otherwise. Y_i is assumed to be Bernoulli distributed,

$$Y_i \sim \text{Bernoulli}(\pi_i) ,$$

with probability π_i. In logistic regression, the logit link function is used to relate the underlying probability π_i to a linear function of the predictors,

$$\text{logit}(\pi_i) = \log\left(\frac{\pi_i}{1-\pi_i}\right) = x_i'\beta ,$$

where x_i is the vector of covariates and β is a vector of regression coefficients. Maximum likelihood estimation is used to estimate the parameters in the model.

Logistic regression in case-cohort studies applied directly to the cases and the subcohort noncases gives asymptotic inference of odds ratios.[3]

Logistic regression models were fitted using the glm package in R. In the simulations, genotype and baseline age were included as covariates in the model. That is,

$$x_i'\beta = \beta_A A_i + \beta_G G_i ,$$

where A_i is age measured in years for the i-th individual; G_i is genotype for the i-th individual coded as 0, 1 or 2 according to the number of risk alleles; β_A is the log(odds ratio) for a one

year increase in age; and β_G is the log(odds ratio) for a one allele increase in the risk allele. In the applied example, the covariates included in the logistic regression models were the genotype of the genetic variant (coded as 0, 1 or 2 depending on the number of risk alleles per individual), age (in years), sex, EPIC-CVD centre (as a categorical variable), and the first ten prinicipal components of ancestry.

Cox regression

Cox proportional hazards models are regularly used to analyse time-to-event data in prospective epidemiological cohort studies.[4] Let $h_i(t)$ be the hazard (or rate) function for the i-th individual, then the Cox model takes the form,

$$h_i(t) = h_0(t)\exp\left(x_i'\beta\right) ,$$

where x_i is the vector of covariates, β is a vector of regression coefficients, and $h_0(t)$ is the baseline hazard function at time t (i.e. when $x = 0$). A partial likelihood is used to fit the Cox model, where the contribution of an event to the likelihood by person i at time t_j is

$$\frac{Z_i(t_j)\exp\left(x_i'\beta\right)}{\sum_{k=1}^{n} Z_k(t_j)\exp\left(x_k'\beta\right)} ,$$

where Z_i is an indicator function denoting censoring that takes the value 1 at times when the i-th subject is at risk and 0 otherwise, and n is the total number of individuals in the cohort.

The Cox model also tends to be the analysis model of choice for case-cohort studies. However, instead of fitting the model with a partial likelihood function the model is fitted using a pseudolikelihood.[3] This pseudolikelihood is weighted to account for the sampling process.

The contribution of an event to the likelihood by person i at time t_j is

$$\frac{Z_i(t_j)w_i(t_j)\exp{(x_i'\beta)}}{Z_i(t_j)w_i(t_j)\exp{(x_i'\beta)} + \sum\limits_{\substack{k\in S\\k\neq i}} Z_k(t_j)w_k(t_j)\exp{(x_k'\beta)}},$$

where Z_i is an indicator function denoting censoring that takes the value 1 at times when the i-th subject is at risk and 0 otherwise, w_i is the weight for the i-th subject, and S refers to the subcohort members. The first term in the denominator is the contribution by the case and the second term is the summation over the subcohort members at risk at the event time. Several weighting strategies have been proposed. The weighting method that yields effect estimates that most closely resemble those from the full cohort are Prentice weights.[5] Prentice weights give the same weight (of 1) to all subcohort members who had not experienced the event before the current event time (cases and noncases in the subcohort). Cases from outside the subcohort are unweighted until their event time when they are given the same weight as the subcohort members. In practice, Prentice weights are equivalent to setting the entry time of the cases from outside the subcohort to just before their event time (e.g. event time - 1×10^{-8}). Since the score contributions to the psuedolikelihood are not independent, robust standard errors (SEs) are necessary.[3,6]

Cox models were fitted using the survival package in R, and the time-on-study time-scale was used for both the simulations and the applied example. Prentice weights and robust SEs were used for the Cox model in the case-cohort setting to account for the sampling process. The Prentice weights were fitted by setting the time-at-entry of the cases from outside the subcohort to just before their event time (event time - 1×10^{-8}); the subcohort members entry times were all set to 0. The same covariates were used in the Cox models as in the logistic regression models in both the simulations and in the applied example (see above).

Supplementary Tables and Figures

Table S1: Type I error rates for simulations of cohort studies with 10,000 individuals.

		Complete		Survey		Random	
Cumulative Disease Incidence	RAF	Cox Regression	Logistic Regression	Cox Regression	Logistic Regression	Cox Regression	Logistic Regression
5%	0.05	0.047	0.047	0.046	0.047	0.048	0.049
	0.10	0.049	0.048	0.047	0.046	0.046	0.047
	0.25	0.054	0.054	0.045	0.043	0.045	0.048
	0.50	0.056	0.056	0.048	0.047	0.046	0.046
	0.75	0.053	0.053	0.045	0.043	0.045	0.048
	0.90	0.048	0.048	0.047	0.046	0.046	0.047
	0.95	0.049	0.049	0.046	0.047	0.048	0.049
10%	0.05	0.044	0.043	0.053	0.055	0.054	0.054
	0.10	0.049	0.048	0.046	0.044	0.051	0.052
	0.25	0.050	0.049	0.049	0.045	0.043	0.044
	0.50	0.052	0.052	0.047	0.049	0.049	0.051
	0.75	0.050	0.050	0.049	0.045	0.043	0.044
	0.90	0.052	0.051	0.046	0.044	0.051	0.052
	0.95	0.052	0.052	0.053	0.055	0.054	0.054
15%	0.05	0.048	0.048	0.054	0.052	0.050	0.047
	0.10	0.050	0.050	0.048	0.046	0.051	0.052
	0.10	0.048	0.049	0.044	0.047	0.052	0.047
	0.10	0.049	0.048	0.047	0.044	0.052	0.053
	0.10	0.051	0.053	0.044	0.047	0.052	0.047
	0.10	0.046	0.046	0.048	0.046	0.051	0.052
	0.10	0.050	0.049	0.054	0.052	0.050	0.047

RAF, risk allele frequency. Complete, survey and random are the types of follow-up over the 20-year period.

Table S2: Type I error rates for simulations of case-cohort studies with sampling fraction of 10% from 40,000 individuals.

		Complete		Survey		Random	
Cumulative Disease Incidence	RAF	Cox Regression	Logistic Regression	Cox Regression	Logistic Regression	Cox Regression	Logistic Regression
5%	0.05	0.051	0.051	0.050	0.048	0.049	0.048
	0.10	0.050	0.049	0.049	0.044	0.047	0.051
	0.25	0.046	0.045	0.045	0.047	0.047	0.044
	0.50	0.046	0.047	0.053	0.049	0.042	0.047
	0.75	0.046	0.045	0.045	0.047	0.047	0.044
	0.90	0.050	0.049	0.049	0.044	0.047	0.051
	0.95	0.051	0.051	0.050	0.048	0.049	0.048
10%	0.05	0.054	0.054	0.052	0.053	0.053	0.047
	0.10	0.052	0.050	0.049	0.047	0.052	0.049
	0.25	0.051	0.053	0.052	0.050	0.050	0.052
	0.50	0.049	0.050	0.052	0.049	0.052	0.050
	0.75	0.051	0.053	0.052	0.050	0.050	0.052
	0.90	0.052	0.050	0.049	0.047	0.052	0.049
	0.95	0.054	0.054	0.052	0.053	0.053	0.047
15%	0.05	0.052	0.057	0.055	0.053	0.052	0.053
	0.10	0.050	0.051	0.054	0.052	0.055	0.049
	0.25	0.047	0.044	0.055	0.051	0.049	0.048
	0.50	0.050	0.051	0.052	0.052	0.047	0.046
	0.75	0.047	0.044	0.055	0.051	0.049	0.048
	0.90	0.050	0.051	0.054	0.052	0.055	0.049
	0.95	0.052	0.057	0.055	0.053	0.052	0.053

RAF, risk allele frequency. Complete, survey and random are the types of follow-up over the 20-year period. The Cox model was Prentice weighted and robust standard errors were used to account for the sampling process.

Table S3: Simulation results for cohort studies with 10,000 individuals for a SNP with RAF=0.10.

	Cox Regression (HR)						Logistic Regression (OR)						
True HR	Mean(Bias)	Mean(SE)	SD(log(HR))	Mean(MSE)	Power	Coverage	Mean(Bias)	Mean(SE)	SD(log(OR))	Mean(MSE)	Power	Coverage	Diff in Power
Complete Follow-up, 5% Cumulative Disease Incidence													
1.00	-0.0035	0.1060	0.1066	0.0226	0.0476	0.9524	-0.0035	0.1091	0.1097	0.0240	0.0482	0.9518	-0.0006
1.05	-0.0023	0.1039	0.1035	0.0215	0.0810	0.9546	-0.0008	0.1070	0.1067	0.0229	0.0820	0.9548	-0.0010
1.10	-0.0039	0.1020	0.1015	0.0207	0.1610	0.9520	-0.0010	0.1052	0.1047	0.0220	0.1590	0.9524	0.0020
1.15	-0.0048	0.1002	0.0994	0.0200	0.2894	0.9538	-0.0005	0.1035	0.1027	0.0213	0.2886	0.9526	0.0008
1.20	-0.0024	0.0985	0.0984	0.0194	0.4674	0.9486	0.0034	0.1018	0.1018	0.0208	0.4636	0.9478	0.0038
1.30	-0.0027	0.0956	0.0947	0.0181	0.7736	0.9534	0.0060	0.0991	0.0982	0.0195	0.7700	0.9518	0.0036
1.50	-0.0031	0.0909	0.0915	0.0166	0.9856	0.9460	0.0111	0.0947	0.0953	0.0182	0.9858	0.9404	-0.0002
2.00	-0.0014	0.0834	0.0836	0.0140	1.0000	0.9466	0.0264	0.0881	0.0882	0.0162	1.0000	0.9334	0.0000
Complete Follow-up, 10% Cumulative Disease Incidence													
1.00	-0.0019	0.0742	0.0747	0.0111	0.0544	0.9456	-0.0019	0.0787	0.0792	0.0125	0.0534	0.9466	0.0010
1.05	-0.0045	0.0729	0.0738	0.0108	0.1008	0.9460	-0.0016	0.0776	0.0785	0.0122	0.1000	0.9460	0.0008
1.10	-0.0025	0.0716	0.0710	0.0102	0.2664	0.9550	0.0036	0.0764	0.0757	0.0116	0.2628	0.9524	0.0036
1.15	-0.0020	0.0704	0.0716	0.0101	0.5100	0.9416	0.0070	0.0753	0.0766	0.0116	0.5078	0.9402	0.0022
1.20	-0.0006	0.0694	0.0700	0.0097	0.7360	0.9468	0.0114	0.0744	0.0750	0.0113	0.7342	0.9418	0.0018
1.30	-0.0003	0.0674	0.0680	0.0092	0.9624	0.9486	0.0176	0.0726	0.0733	0.0110	0.9626	0.9392	-0.0002
1.50	-0.0004	0.0642	0.0633	0.0081	0.9998	0.9544	0.0290	0.0699	0.0689	0.0105	0.9998	0.9278	0.0000
2.00	-0.0005	0.0586	0.0587	0.0069	1.0000	0.9510	0.0576	0.0655	0.0656	0.0119	1.0000	0.8526	0.0000
Complete Follow-up, 15% Cumulative Disease Incidence													
1.00	-0.0011	0.0608	0.0611	0.0074	0.0502	0.9498	-0.0010	0.0666	0.0669	0.0089	0.0498	0.9502	0.0004
1.05	-0.0008	0.0597	0.0591	0.0071	0.1344	0.9534	0.0040	0.0656	0.0650	0.0085	0.1310	0.9524	0.0034
1.10	-0.0008	0.0588	0.0590	0.0069	0.3754	0.9506	0.0087	0.0648	0.0652	0.0085	0.3748	0.9460	0.0006
1.15	-0.0012	0.0578	0.0580	0.0067	0.6660	0.9512	0.0125	0.0640	0.0642	0.0084	0.6596	0.9444	0.0064
1.20	-0.0013	0.0570	0.0576	0.0066	0.8730	0.9490	0.0168	0.0633	0.0638	0.0084	0.8694	0.9390	0.0036
1.30	-0.0005	0.0555	0.0566	0.0063	0.9954	0.9460	0.0268	0.0621	0.0633	0.0086	0.9954	0.9208	0.0000
1.50	-0.0007	0.0530	0.0537	0.0057	1.0000	0.9456	0.0440	0.0601	0.0610	0.0093	1.0000	0.8804	0.0000
2.00	-0.0007	0.0487	0.0482	0.0047	1.0000	0.9520	0.0869	0.0574	0.0567	0.0141	1.0000	0.6768	0.0000
Survey Follow-up, 5% Cumulative Disease Incidence													
1.00	-0.0034	0.1052	0.1045	0.0220	0.0474	0.9526	-0.0036	0.1083	0.1076	0.0233	0.0464	0.9536	0.0010
1.05	-0.0015	0.1031	0.1031	0.0213	0.0818	0.9478	-0.0003	0.1062	0.1065	0.0226	0.0842	0.9466	-0.0024
1.10	-0.0006	0.1011	0.1015	0.0205	0.1678	0.9480	0.0020	0.1044	0.1046	0.0219	0.1684	0.9470	-0.0006
1.15	-0.0037	0.0995	0.1013	0.0202	0.3014	0.9468	-0.0007	0.1028	0.1048	0.0216	0.2938	0.9458	0.0076
1.20	-0.0015	0.0979	0.0958	0.0188	0.4668	0.9576	0.0026	0.1013	0.0991	0.0201	0.4576	0.9572	0.0092
1.30	-0.0043	0.0951	0.0964	0.0184	0.7636	0.9482	0.0022	0.0986	0.1001	0.0198	0.7504	0.9476	0.0132
1.50	-0.0043	0.0907	0.0905	0.0164	0.9864	0.9494	0.0065	0.0945	0.0945	0.0179	0.9858	0.9468	0.0006
2.00	-0.0014	0.0840	0.0829	0.0139	1.0000	0.9534	0.0193	0.0885	0.0878	0.0159	1.0000	0.9418	0.0000
Survey Follow-up, 10% Cumulative Disease Incidence													
1.00	-0.0012	0.0742	0.0741	0.0110	0.0460	0.9540	-0.0011	0.0787	0.0783	0.0123	0.0440	0.9560	0.0020
1.05	-0.0019	0.0728	0.0746	0.0109	0.1148	0.9452	-0.0007	0.0775	0.0792	0.0123	0.1070	0.9458	0.0078
1.10	-0.0035	0.0716	0.0726	0.0104	0.2638	0.9478	-0.0006	0.0763	0.0774	0.0118	0.2550	0.9468	0.0088
1.15	-0.0035	0.0705	0.0705	0.0100	0.4994	0.9506	0.0019	0.0753	0.0755	0.0114	0.4828	0.9494	0.0166
1.20	-0.0016	0.0694	0.0700	0.0097	0.7296	0.9526	0.0057	0.0743	0.0750	0.0112	0.7068	0.9492	0.0228
1.30	-0.0006	0.0675	0.0683	0.0092	0.9638	0.9486	0.0106	0.0726	0.0731	0.0107	0.9556	0.9440	0.0082

1.50	-0.0019	0.0645	0.0635	0.0082	1.0000	0.9530	0.0166	0.0700	0.0691	0.0100	1.0000	0.9448	0.0000
2.00	0.0002	0.0594	0.0598	0.0071	1.0000	0.9518	0.0362	0.0661	0.0659	0.0100	1.0000	0.9160	0.0000
Survey Follow-up, 15% Cumulative Disease Incidence													
1.00	-0.0005	0.0610	0.0605	0.0074	0.0482	0.9518	-0.0006	0.0667	0.0660	0.0088	0.0456	0.9544	0.0026
1.05	-0.0008	0.0599	0.0599	0.0072	0.1300	0.9484	0.0016	0.0658	0.0659	0.0087	0.1226	0.9492	0.0074
1.10	-0.0018	0.0590	0.0585	0.0069	0.3672	0.9522	0.0027	0.0650	0.0639	0.0083	0.3370	0.9550	0.0302
1.15	-0.0007	0.0581	0.0587	0.0068	0.6674	0.9530	0.0062	0.0642	0.0648	0.0084	0.6216	0.9514	0.0458
1.20	-0.0005	0.0573	0.0581	0.0067	0.8760	0.9482	0.0093	0.0635	0.0642	0.0082	0.8472	0.9448	0.0288
1.30	-0.0005	0.0558	0.0571	0.0064	0.9944	0.9428	0.0131	0.0623	0.0639	0.0081	0.9894	0.9406	0.0050
1.50	-0.0011	0.0535	0.0533	0.0057	1.0000	0.9494	0.0219	0.0605	0.0600	0.0077	1.0000	0.9362	0.0000
2.00	-0.0013	0.0498	0.0496	0.0049	1.0000	0.9492	0.0439	0.0580	0.0585	0.0087	1.0000	0.8720	0.0000
Random Follow-up, 5% Cumulative Disease Incidence													
1.00	-0.0013	0.1056	0.1049	0.0222	0.0462	0.9538	-0.0010	0.1087	0.1077	0.0234	0.0474	0.9526	-0.0012
1.05	-0.0066	0.1038	0.1055	0.0220	0.0806	0.9486	-0.0061	0.1069	0.1086	0.0233	0.0790	0.9484	0.0016
1.10	-0.0038	0.1017	0.1032	0.0210	0.1682	0.9466	-0.0022	0.1049	0.1062	0.0223	0.1620	0.9474	0.0062
1.15	-0.0046	0.1000	0.0992	0.0199	0.2864	0.9542	-0.0021	0.1032	0.1025	0.0212	0.2812	0.9552	0.0052
1.20	-0.0025	0.0983	0.0982	0.0193	0.4638	0.9540	0.0004	0.1016	0.1013	0.0206	0.4574	0.9534	0.0064
1.30	0.0010	0.0954	0.0954	0.0182	0.7784	0.9488	0.0056	0.0989	0.0989	0.0196	0.7624	0.9496	0.0160
1.50	-0.0031	0.0913	0.0908	0.0166	0.9858	0.9532	0.0049	0.0950	0.0944	0.0180	0.9836	0.9522	0.0022
2.00	-0.0037	0.0852	0.0858	0.0147	1.0000	0.9504	0.0106	0.0896	0.0902	0.0163	1.0000	0.9448	0.0000
Random Follow-up, 10% Cumulative Disease Incidence													
1.00	-0.0026	0.0747	0.0750	0.0112	0.0508	0.9492	-0.0032	0.0792	0.0793	0.0126	0.0524	0.9476	-0.0016
1.05	-0.0008	0.0733	0.0746	0.0109	0.1146	0.9466	0.0000	0.0779	0.0794	0.0124	0.1096	0.9494	0.0050
1.10	-0.0018	0.0721	0.0727	0.0105	0.2702	0.9486	0.0005	0.0768	0.0775	0.0119	0.2494	0.9456	0.0208
1.15	-0.0005	0.0709	0.0715	0.0101	0.5032	0.9488	0.0023	0.0757	0.0761	0.0115	0.4696	0.9490	0.0336
1.20	-0.0025	0.0699	0.0696	0.0097	0.7324	0.9486	0.0017	0.0748	0.0745	0.0112	0.6898	0.9496	0.0426
1.30	-0.0024	0.0681	0.0675	0.0092	0.9604	0.9542	0.0039	0.0732	0.0725	0.0106	0.9496	0.9508	0.0108
1.50	-0.0029	0.0651	0.0656	0.0086	1.0000	0.9490	0.0078	0.0706	0.0718	0.0102	1.0000	0.9466	0.0000
2.00	-0.0006	0.0604	0.0603	0.0073	1.0000	0.9494	0.0198	0.0668	0.0668	0.0093	1.0000	0.9358	0.0000
Random Follow-up, 15% Cumulative Disease Incidence													
1.00	0.0003	0.0608	0.0617	0.0075	0.0506	0.9494	0.0007	0.0665	0.0674	0.0090	0.0516	0.9484	-0.0010
1.05	-0.0027	0.0598	0.0606	0.0073	0.1274	0.9488	-0.0014	0.0657	0.0661	0.0087	0.1190	0.9460	0.0084
1.10	-0.0016	0.0589	0.0589	0.0069	0.3614	0.9502	-0.0003	0.0648	0.0649	0.0084	0.3240	0.9464	0.0374
1.15	-0.0002	0.0580	0.0579	0.0067	0.6650	0.9484	0.0027	0.0641	0.0642	0.0082	0.6076	0.9494	0.0574
1.20	-0.0012	0.0572	0.0571	0.0065	0.8786	0.9538	0.0031	0.0634	0.0629	0.0080	0.8282	0.9520	0.0504
1.30	-0.0003	0.0558	0.0571	0.0064	0.9946	0.9498	0.0056	0.0622	0.0635	0.0079	0.9876	0.9416	0.0070
1.50	-0.0011	0.0536	0.0533	0.0057	1.0000	0.9488	0.0091	0.0605	0.0602	0.0074	1.0000	0.9480	0.0000
2.00	-0.0008	0.0502	0.0502	0.0050	1.0000	0.9462	0.0184	0.0582	0.0584	0.0071	1.0000	0.9368	0.0000

HR, hazard ratio; OR, odds ratio; RAF, risk allele frequency; Diff in Power, difference in power (Cox - logistic); SD, standard deviation; SE, standard error of the logarithm of hazard or odds ratio; MSE, mean squared error ($MSE=Bias^2+SE^2$). Bias refers to either the estimated log(HR) or log(OR) minus the underlying log(HR). It is important to note that HRs and ORs are different measures of comparison and as such ORs are not ?biased? if different from the underlying HR. Coverage refers to the proportion of 95% confidence intervals that contain the underlying association.

Table S4: Simulation results for cohort studies with 10,000 individuals for a SNP with RAF=0.10 changing the amount of censoring.

	Cox Regression (HR)						Logistic Regression (OR)						
True HR	Mean(Bias)	Mean(SE)	SD(log(HR))	Mean(MSE)	Power	Coverage	Mean(Bias)	Mean(SE)	SD(log(OR))	Mean(MSE)	Power	Coverage	Diff in Power
No Censoring, 5% Cumulative Disease Incidence													
1.00	-0.0035	0.1060	0.1066	0.0226	0.0476	0.9524	-0.0035	0.1091	0.1097	0.0240	0.0482	0.9518	-0.0006
1.05	-0.0023	0.1039	0.1035	0.0215	0.0810	0.9546	-0.0008	0.1070	0.1067	0.0229	0.0820	0.9548	-0.0010
1.10	-0.0039	0.1020	0.1015	0.0207	0.1610	0.9520	-0.0010	0.1052	0.1047	0.0220	0.1590	0.9524	0.0020
1.15	-0.0048	0.1002	0.0994	0.0200	0.2894	0.9538	-0.0005	0.1035	0.1027	0.0213	0.2886	0.9526	0.0008
1.20	-0.0024	0.0985	0.0984	0.0194	0.4674	0.9486	0.0034	0.1018	0.1018	0.0208	0.4636	0.9478	0.0038
1.30	-0.0027	0.0956	0.0947	0.0181	0.7736	0.9534	0.0060	0.0991	0.0982	0.0195	0.7700	0.9518	0.0036
1.50	-0.0031	0.0909	0.0915	0.0166	0.9856	0.9460	0.0111	0.0947	0.0953	0.0182	0.9858	0.9404	-0.0002
2.00	-0.0014	0.0834	0.0836	0.0140	1.0000	0.9466	0.0264	0.0881	0.0882	0.0162	1.0000	0.9334	0.0000
No Censoring, 10% Cumulative Disease Incidence													
1.00	-0.0019	0.0742	0.0747	0.0111	0.0544	0.9456	-0.0019	0.0787	0.0792	0.0125	0.0534	0.9466	0.0010
1.05	-0.0045	0.0729	0.0738	0.0108	0.1008	0.9460	-0.0016	0.0776	0.0785	0.0122	0.1000	0.9460	0.0008
1.10	-0.0025	0.0716	0.0710	0.0102	0.2664	0.9550	0.0036	0.0764	0.0757	0.0116	0.2628	0.9524	0.0036
1.15	-0.0020	0.0704	0.0716	0.0101	0.5100	0.9416	0.0070	0.0753	0.0766	0.0116	0.5078	0.9402	0.0022
1.20	-0.0006	0.0694	0.0700	0.0097	0.7360	0.9468	0.0114	0.0744	0.0750	0.0113	0.7342	0.9418	0.0018
1.30	-0.0003	0.0674	0.0680	0.0092	0.9624	0.9486	0.0176	0.0726	0.0733	0.0110	0.9626	0.9392	-0.0002
1.50	-0.0004	0.0642	0.0633	0.0081	0.9998	0.9544	0.0290	0.0699	0.0689	0.0105	0.9998	0.9278	0.0000
2.00	-0.0005	0.0586	0.0587	0.0069	1.0000	0.9510	0.0576	0.0655	0.0656	0.0119	1.0000	0.8526	0.0000
No Censoring, 15% Cumulative Disease Incidence													
1.00	-0.0011	0.0608	0.0611	0.0074	0.0502	0.9498	-0.0010	0.0666	0.0669	0.0089	0.0498	0.9502	0.0004
1.05	-0.0008	0.0597	0.0591	0.0071	0.1344	0.9534	0.0040	0.0656	0.0650	0.0085	0.1310	0.9524	0.0034
1.10	-0.0008	0.0588	0.0590	0.0069	0.3754	0.9506	0.0087	0.0648	0.0652	0.0085	0.3748	0.9460	0.0006
1.15	-0.0012	0.0578	0.0580	0.0067	0.6660	0.9512	0.0125	0.0640	0.0642	0.0084	0.6596	0.9444	0.0064
1.20	-0.0013	0.0570	0.0576	0.0066	0.8730	0.9490	0.0168	0.0633	0.0638	0.0084	0.8694	0.9390	0.0036
1.30	-0.0005	0.0555	0.0566	0.0063	0.9954	0.9460	0.0268	0.0621	0.0633	0.0086	0.9954	0.9208	0.0000
1.50	-0.0007	0.0530	0.0537	0.0057	1.0000	0.9456	0.0440	0.0601	0.0610	0.0093	1.0000	0.8804	0.0000
2.00	-0.0007	0.0487	0.0482	0.0047	1.0000	0.9520	0.0869	0.0574	0.0567	0.0141	1.0000	0.6768	0.0000
Survey Censoring, 5% Cumulative Disease Incidence (if there was no censoring)													
1.00	-0.0056	0.1333	0.1327	0.0355	0.0480	0.9520	-0.0056	0.1357	0.1350	0.0367	0.0474	0.9526	0.0006
1.05	-0.0048	0.1305	0.1299	0.0340	0.0698	0.9522	-0.0038	0.1330	0.1322	0.0352	0.0680	0.9516	0.0018
1.10	-0.0091	0.1282	0.1300	0.0335	0.1250	0.9480	-0.0076	0.1307	0.1325	0.0348	0.1226	0.9480	0.0024
1.15	-0.0052	0.1257	0.1243	0.0313	0.2046	0.9530	-0.0030	0.1283	0.1267	0.0326	0.2046	0.9542	0.0000
1.20	-0.0023	0.1234	0.1234	0.0305	0.3322	0.9492	0.0006	0.1261	0.1262	0.0319	0.3308	0.9482	0.0014
1.30	-0.0046	0.1196	0.1196	0.0287	0.5894	0.9540	-0.0005	0.1224	0.1219	0.0299	0.5842	0.9536	0.0052
1.50	-0.0027	0.1133	0.1122	0.0255	0.9330	0.9548	0.0047	0.1163	0.1155	0.0269	0.9298	0.9532	0.0032
2.00	-0.0032	0.1032	0.1031	0.0213	1.0000	0.9512	0.0116	0.1069	0.1069	0.0230	1.0000	0.9510	0.0000
Survey Censoring, 10% Cumulative Disease Incidence (if there was no censoring)													
1.00	0.0003	0.0967	0.0974	0.0189	0.0516	0.9484	0.0008	0.1001	0.1008	0.0202	0.0548	0.9452	-0.0032
1.05	-0.0061	0.0951	0.0953	0.0182	0.0832	0.9504	-0.0051	0.0986	0.0988	0.0195	0.0862	0.9518	-0.0030
1.10	-0.0044	0.0934	0.0945	0.0177	0.1816	0.9470	-0.0018	0.0969	0.0981	0.0190	0.1766	0.9458	0.0050
1.15	-0.0023	0.0917	0.0920	0.0169	0.3460	0.9540	0.0017	0.0953	0.0957	0.0183	0.3440	0.9516	0.0020

1.20	-0.0039	0.0903	0.0907	0.0164	0.5180	0.9494	0.0009	0.0940	0.0943	0.0177	0.5004	0.9498	0.0176
1.30	-0.0018	0.0876	0.0875	0.0153	0.8358	0.9514	0.0052	0.0914	0.0913	0.0167	0.8272	0.9502	0.0086
1.50	-0.0013	0.0831	0.0839	0.0140	0.9958	0.9468	0.0110	0.0872	0.0884	0.0155	0.9948	0.9420	0.0010
2.00	-0.0015	0.0753	0.0767	0.0116	1.0000	0.9468	0.0229	0.0804	0.0819	0.0137	1.0000	0.9316	0.0000
Survey Censoring, 15% Cumulative Disease Incidence (if there was no censoring)													
1.00	-0.0011	0.0808	0.0803	0.0130	0.0468	0.9532	-0.0012	0.0849	0.0844	0.0143	0.0474	0.9526	-0.0006
1.05	-0.0017	0.0793	0.0788	0.0125	0.0986	0.9526	-0.0001	0.0835	0.0830	0.0139	0.0934	0.9526	0.0052
1.10	-0.0014	0.0779	0.0786	0.0123	0.2402	0.9506	0.0015	0.0822	0.0828	0.0136	0.2334	0.9508	0.0068
1.15	-0.0029	0.0767	0.0761	0.0117	0.4446	0.9498	0.0015	0.0811	0.0805	0.0131	0.4282	0.9510	0.0164
1.20	-0.0018	0.0755	0.0749	0.0113	0.6648	0.9536	0.0049	0.0800	0.0792	0.0127	0.6430	0.9532	0.0218
1.30	-0.0007	0.0733	0.0735	0.0108	0.9308	0.9496	0.0088	0.0779	0.0782	0.0123	0.9238	0.9498	0.0070
1.50	-0.0035	0.0697	0.0704	0.0098	0.9992	0.9484	0.0131	0.0748	0.0754	0.0115	0.9990	0.9440	0.0002
2.00	-0.0002	0.0634	0.0642	0.0082	1.0000	0.9518	0.0324	0.0696	0.0707	0.0109	1.0000	0.9144	0.0000
Random Follow-up, 5% Cumulative Disease Incidence (if there was no censoring)													
1.00	-0.0069	0.1488	0.1482	0.0443	0.0482	0.9518	-0.0069	0.1509	0.1505	0.0456	0.0486	0.9514	-0.0004
1.05	-0.0092	0.1460	0.1458	0.0427	0.0666	0.9506	-0.0092	0.1481	0.1484	0.0441	0.0660	0.9510	0.0006
1.10	-0.0116	0.1430	0.1429	0.0411	0.1086	0.9524	-0.0107	0.1453	0.1451	0.0424	0.1108	0.9522	-0.0022
1.15	-0.0058	0.1402	0.1424	0.0400	0.1918	0.9508	-0.0042	0.1425	0.1449	0.0414	0.1926	0.9490	-0.0008
1.20	-0.0031	0.1377	0.1386	0.0382	0.2846	0.9528	-0.0010	0.1400	0.1410	0.0396	0.2840	0.9528	0.0006
1.30	-0.0064	0.1333	0.1316	0.0352	0.4980	0.9564	-0.0031	0.1358	0.1343	0.0365	0.4938	0.9556	0.0042
1.50	-0.0067	0.1262	0.1272	0.0322	0.8644	0.9506	-0.0014	0.1288	0.1298	0.0335	0.8590	0.9488	0.0054
2.00	-0.0032	0.1145	0.1134	0.0260	1.0000	0.9542	0.0071	0.1178	0.1168	0.0276	1.0000	0.9532	0.0000
Random Censoring, 10% Cumulative Disease Incidence (if there was no censoring)													
1.00	-0.0036	0.1097	0.1086	0.0238	0.0450	0.9550	-0.0041	0.1126	0.1117	0.0252	0.0456	0.9544	-0.0006
1.05	-0.0045	0.1075	0.1074	0.0231	0.0754	0.9546	-0.0037	0.1105	0.1102	0.0244	0.0768	0.9546	-0.0014
1.10	-0.0052	0.1056	0.1050	0.0222	0.1502	0.9514	-0.0039	0.1087	0.1079	0.0235	0.1506	0.9508	-0.0004
1.15	-0.0027	0.1037	0.1031	0.0214	0.2804	0.9554	-0.0009	0.1069	0.1060	0.0227	0.2732	0.9536	0.0072
1.20	-0.0039	0.1021	0.1032	0.0211	0.4324	0.9502	-0.0010	0.1053	0.1062	0.0224	0.4206	0.9488	0.0118
1.30	-0.0026	0.0989	0.0996	0.0197	0.7364	0.9486	0.0022	0.1023	0.1029	0.0211	0.7234	0.9464	0.0130
1.50	-0.0043	0.0938	0.0930	0.0175	0.9844	0.9542	0.0029	0.0975	0.0963	0.0188	0.9830	0.9546	0.0014
2.00	0.0003	0.0846	0.0850	0.0144	1.0000	0.9494	0.0161	0.0891	0.0893	0.0162	1.0000	0.9424	0.0000
Random Censoring, 15% Cumulative Disease Incidence (if there was no censoring)													
1.00	-0.0010	0.0920	0.0910	0.0168	0.0480	0.9520	-0.0011	0.0956	0.0945	0.0181	0.0494	0.9508	-0.0014
1.05	-0.0038	0.0903	0.0893	0.0162	0.0832	0.9554	-0.0029	0.0940	0.0928	0.0175	0.0802	0.9538	0.0030
1.10	-0.0014	0.0887	0.0885	0.0157	0.2022	0.9506	0.0008	0.0924	0.0920	0.0170	0.1950	0.9508	0.0072
1.15	-0.0033	0.0872	0.0878	0.0153	0.3664	0.9490	-0.0002	0.0910	0.0918	0.0167	0.3506	0.9494	0.0158
1.20	-0.0025	0.0859	0.0862	0.0148	0.5582	0.9530	0.0007	0.0898	0.0902	0.0162	0.5380	0.9518	0.0202
1.30	-0.0012	0.0833	0.0838	0.0140	0.8660	0.9514	0.0042	0.0874	0.0882	0.0154	0.8470	0.9488	0.0190
1.50	-0.0036	0.0792	0.0805	0.0128	0.9962	0.9476	0.0050	0.0836	0.0846	0.0142	0.9956	0.9470	0.0006
2.00	-0.0020	0.0719	0.0724	0.0104	1.0000	0.9464	0.0162	0.0771	0.0774	0.0122	1.0000	0.9398	0.0000

HR, hazard ratio; OR, odds ratio; RAF, risk allele frequency; Diff in Power, difference in power (Cox - logistic); SD, standard deviation; SE, standard error of the logarithm of hazard or odds ratio; MSE, mean squared error (MSE=$Bias^2+SE^2$). Bias refers to either the estimated log(HR) or log(OR) minus the underlying log(HR). It is important to note that HRs and ORs are different measures of comparison and as such ORs are not ?biased? if different from the underlying HR. Coverage refers to the proportion of 95% confidence intervals that contain the underlying association.

Table S5: Simulation results for case-cohort studies with sampling fraction of 10% from 40,000 individuals for a SNP with RAF=0.10.

	Cox Regression (HR)						Logistic Regression (OR)						
True HR	Mean(Bias)	Mean(SE)	SD(log(HR))	Mean(MSE)	Power	Coverage	Mean(Bias)	Mean(SE)	SD(log(OR))	Mean(MSE)	Power	Coverage	Diff in Power
Complete Follow-up, 5% Cumulative Disease Incidence													
1.00	-0.0011	0.0651	0.0646	0.0084	0.0500	0.9500	-0.0012	0.0662	0.0658	0.0087	0.0494	0.9506	0.0006
1.05	-0.0004	0.0642	0.0648	0.0083	0.1216	0.9500	0.0008	0.0654	0.0661	0.0086	0.1224	0.9486	-0.0008
1.10	-0.0007	0.0635	0.0642	0.0082	0.3284	0.9490	0.0021	0.0647	0.0656	0.0085	0.3318	0.9490	-0.0034
1.15	0.0005	0.0628	0.0637	0.0080	0.6104	0.9484	0.0046	0.0640	0.0651	0.0084	0.6150	0.9466	-0.0046
1.20	-0.0002	0.0622	0.0618	0.0077	0.8308	0.9496	0.0053	0.0634	0.0629	0.0080	0.8392	0.9524	-0.0084
1.30	-0.0004	0.0612	0.0609	0.0075	0.9896	0.9514	0.0076	0.0624	0.0622	0.0078	0.9904	0.9496	-0.0008
1.50	0.0010	0.0596	0.0599	0.0071	1.0000	0.9482	0.0143	0.0608	0.0608	0.0076	1.0000	0.9424	0.0000
2.00	0.0007	0.0582	0.0580	0.0068	1.0000	0.9496	0.0259	0.0588	0.0588	0.0076	1.0000	0.9280	0.0000
Complete Follow-up, 10% Cumulative Disease Incidence													
1.00	0.0000	0.0532	0.0540	0.0057	0.0524	0.9476	0.0001	0.0549	0.0557	0.0061	0.0498	0.9502	0.0026
1.05	-0.0002	0.0527	0.0526	0.0055	0.1446	0.9486	0.0026	0.0545	0.0544	0.0059	0.1482	0.9454	-0.0036
1.10	0.0001	0.0524	0.0537	0.0056	0.4480	0.9480	0.0054	0.0542	0.0553	0.0060	0.4580	0.9472	-0.0100
1.15	0.0003	0.0520	0.0525	0.0055	0.7630	0.9480	0.0087	0.0539	0.0545	0.0059	0.7822	0.9456	-0.0192
1.20	0.0006	0.0517	0.0523	0.0054	0.9430	0.9480	0.0119	0.0536	0.0541	0.0059	0.9522	0.9436	-0.0092
1.30	0.0003	0.0512	0.0511	0.0052	0.9996	0.9508	0.0166	0.0531	0.0531	0.0059	0.9996	0.9420	0.0000
1.50	-0.0001	0.0505	0.0507	0.0051	1.0000	0.9526	0.0270	0.0524	0.0524	0.0062	1.0000	0.9228	0.0000
2.00	0.0004	0.0503	0.0504	0.0051	1.0000	0.9506	0.0531	0.0518	0.0517	0.0082	1.0000	0.8274	0.0000
Complete Follow-up, 15% Cumulative Disease Incidence													
1.00	0.0001	0.0490	0.0499	0.0049	0.0498	0.9502	0.0005	0.0514	0.0521	0.0054	0.0510	0.9490	-0.0012
1.05	0.0014	0.0487	0.0490	0.0048	0.1784	0.9462	0.0058	0.0512	0.0515	0.0053	0.1848	0.9476	-0.0064
1.10	0.0011	0.0484	0.0480	0.0047	0.5126	0.9534	0.0098	0.0510	0.0506	0.0053	0.5402	0.9504	-0.0276
1.15	0.0005	0.0482	0.0474	0.0046	0.8390	0.9516	0.0132	0.0509	0.0506	0.0053	0.8594	0.9434	-0.0204
1.20	-0.0008	0.0481	0.0485	0.0047	0.9674	0.9502	0.0161	0.0507	0.0513	0.0055	0.9782	0.9398	-0.0108
1.30	-0.0002	0.0478	0.0482	0.0046	0.9998	0.9466	0.0253	0.0505	0.0513	0.0058	1.0000	0.9172	-0.0002
1.50	0.0003	0.0475	0.0479	0.0045	1.0000	0.9540	0.0418	0.0504	0.0503	0.0068	1.0000	0.8730	0.0000
2.00	0.0022	0.0479	0.0476	0.0046	1.0000	0.9468	0.0816	0.0507	0.0503	0.0117	1.0000	0.6368	0.0000
Survey Follow-up, 5% Cumulative Disease Incidence													
1.00	0.0011	0.0674	0.0673	0.0091	0.0490	0.9510	0.0002	0.0659	0.0653	0.0086	0.0444	0.9556	0.0046
1.05	-0.0003	0.0667	0.0669	0.0089	0.1182	0.9482	0.0011	0.0651	0.0653	0.0085	0.1256	0.9488	-0.0074
1.10	0.0002	0.0659	0.0657	0.0087	0.3120	0.9534	0.0027	0.0643	0.0638	0.0082	0.3378	0.9530	-0.0258
1.15	0.0001	0.0652	0.0651	0.0085	0.5668	0.9510	0.0028	0.0637	0.0638	0.0081	0.5982	0.9504	-0.0314
1.20	0.0008	0.0647	0.0643	0.0083	0.8050	0.9538	0.0049	0.0631	0.0629	0.0080	0.8402	0.9542	-0.0352
1.30	0.0011	0.0637	0.0638	0.0081	0.9836	0.9484	0.0069	0.0621	0.0621	0.0078	0.9898	0.9454	-0.0062
1.50	-0.0002	0.0624	0.0631	0.0079	1.0000	0.9462	0.0094	0.0607	0.0611	0.0075	1.0000	0.9406	0.0000
2.00	0.0015	0.0612	0.0614	0.0075	1.0000	0.9482	0.0195	0.0590	0.0588	0.0073	1.0000	0.9402	0.0000
Survey Follow-up, 10% Cumulative Disease Incidence													
1.00	-0.0002	0.0577	0.0577	0.0067	0.0494	0.9506	-0.0007	0.0549	0.0547	0.0060	0.0470	0.9530	0.0024
1.05	0.0010	0.0572	0.0572	0.0066	0.1324	0.9474	0.0023	0.0545	0.0550	0.0060	0.1550	0.9482	-0.0226
1.10	-0.0004	0.0569	0.0570	0.0065	0.3786	0.9518	0.0026	0.0541	0.0547	0.0059	0.4350	0.9478	-0.0564
1.15	0.0018	0.0566	0.0569	0.0064	0.7034	0.9516	0.0069	0.0538	0.0538	0.0058	0.7814	0.9484	-0.0780

1.20	0.0009	0.0563	0.0564	0.0063	0.9038	0.9490	0.0076	0.0536	0.0538	0.0058	0.9422	0.9482	-0.0384
1.30	-0.0011	0.0558	0.0555	0.0062	0.9972	0.9534	0.0094	0.0531	0.0528	0.0057	0.9996	0.9476	-0.0024
1.50	0.0007	0.0553	0.0554	0.0061	1.0000	0.9530	0.0171	0.0525	0.0527	0.0058	1.0000	0.9396	0.0000
2.00	0.0024	0.0551	0.0556	0.0061	1.0000	0.9496	0.0334	0.0519	0.0522	0.0065	1.0000	0.9034	0.0000
Survey Follow-up, 15% Cumulative Disease Incidence													
1.00	-0.0006	0.0547	0.0556	0.0061	0.0544	0.9456	-0.0004	0.0514	0.0516	0.0053	0.0516	0.9484	0.0028
1.05	0.0015	0.0544	0.0550	0.0060	0.1492	0.9470	0.0028	0.0512	0.0525	0.0054	0.1704	0.9422	-0.0212
1.10	0.0019	0.0542	0.0552	0.0060	0.4332	0.9480	0.0064	0.0510	0.0514	0.0053	0.5130	0.9488	-0.0798
1.15	0.0007	0.0540	0.0548	0.0059	0.7398	0.9478	0.0066	0.0508	0.0510	0.0052	0.8258	0.9478	-0.0860
1.20	-0.0005	0.0538	0.0538	0.0058	0.9294	0.9474	0.0089	0.0507	0.0504	0.0052	0.9682	0.9508	-0.0388
1.30	0.0007	0.0536	0.0544	0.0058	0.9984	0.9484	0.0141	0.0505	0.0513	0.0054	0.9996	0.9408	-0.0012
1.50	0.0016	0.0533	0.0536	0.0057	1.0000	0.9484	0.0230	0.0504	0.0506	0.0056	1.0000	0.9252	0.0000
2.00	0.0008	0.0535	0.0542	0.0058	1.0000	0.9496	0.0424	0.0506	0.0508	0.0069	1.0000	0.8690	0.0000
Random Follow-up, 5% Cumulative Disease Incidence													
1.00	0.0005	0.0696	0.0700	0.0097	0.0468	0.9494	0.0005	0.0661	0.0661	0.0087	0.0506	0.9494	-0.0038
1.05	0.0020	0.0688	0.0699	0.0096	0.1196	0.9450	0.0022	0.0653	0.0660	0.0086	0.1268	0.9450	-0.0072
1.10	-0.0007	0.0681	0.0677	0.0092	0.2864	0.9504	-0.0001	0.0645	0.0641	0.0083	0.3212	0.9504	-0.0348
1.15	0.0008	0.0675	0.0677	0.0091	0.5534	0.9464	0.0028	0.0639	0.0642	0.0082	0.6038	0.9464	-0.0504
1.20	0.0005	0.0670	0.0664	0.0089	0.7768	0.9528	0.0030	0.0633	0.0625	0.0079	0.8334	0.9528	-0.0566
1.30	-0.0005	0.0660	0.0653	0.0086	0.9788	0.9548	0.0038	0.0623	0.0613	0.0076	0.9888	0.9548	-0.0100
1.50	-0.0005	0.0648	0.0648	0.0084	1.0000	0.9440	0.0067	0.0609	0.0616	0.0075	1.0000	0.9440	0.0000
2.00	0.0007	0.0639	0.0649	0.0083	1.0000	0.9370	0.0143	0.0593	0.0604	0.0074	1.0000	0.9370	0.0000
Random Follow-up, 10% Cumulative Disease Incidence													
1.00	-0.0002	0.0606	0.0608	0.0074	0.0524	0.9506	-0.0006	0.0550	0.0547	0.0060	0.0494	0.9506	0.0030
1.05	0.0006	0.0602	0.0601	0.0072	0.1250	0.9494	0.0013	0.0546	0.0547	0.0060	0.1504	0.9494	-0.0254
1.10	0.0006	0.0599	0.0598	0.0072	0.3536	0.9516	0.0021	0.0543	0.0539	0.0059	0.4290	0.9516	-0.0754
1.15	-0.0004	0.0595	0.0591	0.0070	0.6530	0.9520	0.0023	0.0540	0.0537	0.0058	0.7522	0.9520	-0.0992
1.20	-0.0005	0.0593	0.0583	0.0069	0.8756	0.9558	0.0037	0.0537	0.0526	0.0057	0.9398	0.9558	-0.0642
1.30	0.0028	0.0589	0.0590	0.0070	0.9948	0.9502	0.0073	0.0532	0.0533	0.0057	0.9992	0.9502	-0.0044
1.50	0.0004	0.0583	0.0589	0.0069	1.0000	0.9456	0.0097	0.0526	0.0528	0.0057	1.0000	0.9456	0.0000
2.00	0.0004	0.0583	0.0581	0.0068	1.0000	0.9400	0.0192	0.0520	0.0516	0.0057	1.0000	0.9400	0.0000
Random Follow-up, 15% Cumulative Disease Incidence													
1.00	0.0007	0.0579	0.0586	0.0068	0.0552	0.9512	0.0003	0.0513	0.0518	0.0053	0.0488	0.9512	0.0064
1.05	0.0013	0.0577	0.0575	0.0066	0.1366	0.9470	0.0016	0.0511	0.0516	0.0053	0.1642	0.9470	-0.0276
1.10	-0.0002	0.0574	0.0577	0.0066	0.3740	0.9508	0.0016	0.0509	0.0507	0.0052	0.4726	0.9508	-0.0986
1.15	0.0003	0.0572	0.0572	0.0065	0.6920	0.9492	0.0035	0.0508	0.0509	0.0052	0.8058	0.9492	-0.1138
1.20	0.0021	0.0571	0.0569	0.0065	0.9062	0.9478	0.0055	0.0507	0.0513	0.0052	0.9578	0.9478	-0.0516
1.30	0.0014	0.0569	0.0562	0.0064	0.9976	0.9542	0.0065	0.0505	0.0500	0.0051	0.9992	0.9542	-0.0016
1.50	0.0004	0.0566	0.0566	0.0064	1.0000	0.9448	0.0112	0.0503	0.0503	0.0052	1.0000	0.9448	0.0000
2.00	0.0031	0.0571	0.0581	0.0066	1.0000	0.9402	0.0199	0.0505	0.0500	0.0055	1.0000	0.9402	0.0000

HR, hazard ratio; OR, odds ratio; RAF, risk allele frequency; Diff in Power, difference in power (Cox - logistic); SD, standard deviation; SE, standard error of the logarithm of hazard or odds ratio; MSE, mean squared error (MSE=Bias^2+SE^2). Bias refers to either the estimated log(HR) or log(OR) minus the underlying log(HR). It is important to note that HRs and ORs are different measures of comparison and as such ORs are not ?biased? if different from the underlying HR. Coverage refers to the proportion of 95% confidence intervals that contain the underlying association. The Cox model was Prentice weighted and robust SEs were used to account for the sampling process.

Table S6: Simulation results for case-cohort studies with sampling fraction of 10% from 40,000 individuals for a SNP with RAF=0.10 changing the amount of censoring.

	Cox Regression (HR)						Logistic Regression (OR)						
True HR	Mean(Bias)	Mean(SE)	SD(log(HR))	Mean(MSE)	Power	Coverage	Mean(Bias)	Mean(SE)	SD(log(OR))	Mean(MSE)	Power	Coverage	Diff in Power
No Censoring, 5% Cumulative Disease Incidence													
1.00	-0.0011	0.0651	0.0646	0.0084	0.0500	0.9500	-0.0012	0.0662	0.0658	0.0087	0.0494	0.9506	0.0006
1.05	-0.0004	0.0642	0.0648	0.0083	0.1216	0.9500	0.0008	0.0654	0.0661	0.0086	0.1224	0.9486	-0.0008
1.10	-0.0007	0.0635	0.0642	0.0082	0.3284	0.9490	0.0021	0.0647	0.0656	0.0085	0.3318	0.9490	-0.0034
1.15	0.0005	0.0628	0.0637	0.0080	0.6104	0.9484	0.0046	0.0640	0.0651	0.0084	0.6150	0.9466	-0.0046
1.20	-0.0002	0.0622	0.0618	0.0077	0.8308	0.9496	0.0053	0.0634	0.0629	0.0080	0.8392	0.9524	-0.0084
1.30	-0.0004	0.0612	0.0609	0.0075	0.9896	0.9514	0.0076	0.0624	0.0622	0.0078	0.9904	0.9496	-0.0008
1.50	0.0010	0.0596	0.0599	0.0071	1.0000	0.9482	0.0143	0.0608	0.0608	0.0076	1.0000	0.9424	0.0000
2.00	0.0007	0.0582	0.0580	0.0068	1.0000	0.9496	0.0259	0.0588	0.0588	0.0076	1.0000	0.9280	0.0000
No Censoring, 10% Cumulative Disease Incidence													
1.00	0.0000	0.0532	0.0540	0.0057	0.0524	0.9476	0.0001	0.0549	0.0557	0.0061	0.0498	0.9502	0.0026
1.05	-0.0002	0.0527	0.0526	0.0055	0.1446	0.9486	0.0026	0.0545	0.0544	0.0059	0.1482	0.9454	-0.0036
1.10	0.0001	0.0524	0.0537	0.0056	0.4480	0.9480	0.0054	0.0542	0.0553	0.0060	0.4580	0.9472	-0.0100
1.15	0.0003	0.0520	0.0525	0.0055	0.7630	0.9480	0.0087	0.0539	0.0545	0.0059	0.7822	0.9456	-0.0192
1.20	0.0006	0.0517	0.0523	0.0054	0.9430	0.9480	0.0119	0.0536	0.0541	0.0059	0.9522	0.9436	-0.0092
1.30	0.0003	0.0512	0.0511	0.0052	0.9996	0.9508	0.0166	0.0531	0.0531	0.0059	0.9996	0.9420	0.0000
1.50	-0.0001	0.0505	0.0507	0.0051	1.0000	0.9526	0.0270	0.0524	0.0524	0.0062	1.0000	0.9228	0.0000
2.00	0.0004	0.0503	0.0504	0.0051	1.0000	0.9506	0.0531	0.0518	0.0517	0.0082	1.0000	0.8274	0.0000
No Censoring, 15% Cumulative Disease Incidence													
1.00	0.0001	0.0490	0.0499	0.0049	0.0498	0.9502	0.0005	0.0514	0.0521	0.0054	0.0510	0.9490	-0.0012
1.05	0.0014	0.0487	0.0490	0.0048	0.1784	0.9462	0.0058	0.0512	0.0515	0.0053	0.1848	0.9476	-0.0064
1.10	0.0011	0.0484	0.0480	0.0047	0.5126	0.9534	0.0098	0.0510	0.0506	0.0053	0.5402	0.9504	-0.0276
1.15	0.0005	0.0482	0.0474	0.0046	0.8390	0.9516	0.0132	0.0509	0.0506	0.0053	0.8594	0.9434	-0.0204
1.20	-0.0008	0.0481	0.0485	0.0047	0.9674	0.9502	0.0161	0.0507	0.0513	0.0055	0.9782	0.9398	-0.0108
1.30	-0.0002	0.0478	0.0482	0.0046	0.9998	0.9466	0.0253	0.0505	0.0513	0.0058	1.0000	0.9172	-0.0002
1.50	0.0003	0.0475	0.0479	0.0045	1.0000	0.9540	0.0418	0.0504	0.0503	0.0068	1.0000	0.8730	0.0000
2.00	0.0022	0.0479	0.0476	0.0046	1.0000	0.9468	0.0816	0.0507	0.0503	0.0117	1.0000	0.6368	0.0000
Survey Censoring, 5% Cumulative Disease Incidence (if there was no censoring)													
1.00	-0.0022	0.0781	0.0781	0.0122	0.0494	0.9506	-0.0023	0.0774	0.0774	0.0120	0.0532	0.9468	-0.0038
1.05	-0.0014	0.0770	0.0762	0.0117	0.0980	0.9528	-0.0010	0.0762	0.0751	0.0114	0.0954	0.9534	0.0026
1.10	-0.0030	0.0759	0.0771	0.0117	0.2398	0.9506	-0.0013	0.0752	0.0760	0.0114	0.2508	0.9494	-0.0110
1.15	-0.0030	0.0750	0.0748	0.0112	0.4592	0.9462	-0.0007	0.0742	0.0741	0.0110	0.4766	0.9498	-0.0174
1.20	-0.0006	0.0742	0.0737	0.0109	0.6898	0.9526	0.0022	0.0733	0.0732	0.0107	0.7182	0.9478	-0.0284
1.30	0.0012	0.0727	0.0729	0.0106	0.9486	0.9476	0.0047	0.0718	0.0719	0.0103	0.9562	0.9508	-0.0076
1.50	-0.0009	0.0703	0.0708	0.0100	0.9998	0.9462	0.0064	0.0694	0.0701	0.0098	1.0000	0.9454	-0.0002
2.00	0.0015	0.0675	0.0671	0.0091	1.0000	0.9520	0.0151	0.0660	0.0650	0.0088	1.0000	0.9478	0.0000
Survey Censoring, 10% Cumulative Disease Incidence (if there was no censoring)													
1.00	0.0003	0.0645	0.0650	0.0084	0.0490	0.9510	-0.0001	0.0627	0.0633	0.0079	0.0504	0.9496	-0.0014
1.05	-0.0004	0.0638	0.0634	0.0081	0.1170	0.9494	0.0003	0.0620	0.0611	0.0076	0.1234	0.9544	-0.0064
1.10	-0.0009	0.0632	0.0623	0.0079	0.3210	0.9530	0.0018	0.0614	0.0605	0.0074	0.3510	0.9522	-0.0300
1.15	-0.0010	0.0626	0.0642	0.0080	0.6064	0.9430	0.0024	0.0608	0.0619	0.0075	0.6522	0.9466	-0.0458

1.20	-0.0005	0.0621	0.0624	0.0078	0.8356	0.9504	0.0041	0.0603	0.0605	0.0073	0.8704	0.9530	-0.0348
1.30	0.0004	0.0613	0.0616	0.0076	0.9904	0.9478	0.0075	0.0594	0.0604	0.0072	0.9926	0.9420	-0.0022
1.50	0.0017	0.0601	0.0611	0.0073	1.0000	0.9466	0.0131	0.0581	0.0591	0.0070	1.0000	0.9428	0.0000
2.00	0.0007	0.0588	0.0593	0.0070	1.0000	0.9488	0.0223	0.0561	0.0564	0.0068	1.0000	0.9272	0.0000
Survey Censoring, 15% Cumulative Disease Incidence (if there was no censoring)													
1.00	-0.0016	0.0595	0.0592	0.0070	0.0476	0.9524	-0.0011	0.0570	0.0564	0.0064	0.0448	0.9552	0.0028
1.05	-0.0003	0.0590	0.0595	0.0070	0.1342	0.9522	0.0011	0.0565	0.0569	0.0064	0.1442	0.9510	-0.0100
1.10	0.0001	0.0586	0.0586	0.0069	0.3678	0.9478	0.0035	0.0561	0.0565	0.0064	0.4202	0.9462	-0.0524
1.15	0.0008	0.0582	0.0587	0.0068	0.6710	0.9498	0.0053	0.0557	0.0561	0.0063	0.7406	0.9466	-0.0696
1.20	0.0002	0.0578	0.0580	0.0067	0.8846	0.9494	0.0060	0.0553	0.0560	0.0062	0.9246	0.9480	-0.0400
1.30	0.0015	0.0572	0.0579	0.0066	0.9960	0.9456	0.0102	0.0547	0.0548	0.0061	0.9994	0.9448	-0.0034
1.50	0.0000	0.0564	0.0568	0.0064	1.0000	0.9502	0.0147	0.0539	0.0546	0.0061	1.0000	0.9382	0.0000
2.00	0.0016	0.0559	0.0565	0.0063	1.0000	0.9536	0.0298	0.0528	0.0527	0.0065	1.0000	0.9168	0.0000
Random Follow-up, 5% Cumulative Disease Incidence (if there was no censoring)													
1.00	-0.0026	0.0858	0.0861	0.0148	0.0498	0.9518	-0.0023	0.0840	0.0839	0.0141	0.0482	0.9518	0.0016
1.05	-0.0019	0.0845	0.0839	0.0142	0.0898	0.9510	-0.0014	0.0826	0.0825	0.0136	0.0942	0.9510	-0.0044
1.10	-0.0019	0.0832	0.0850	0.0142	0.2174	0.9464	-0.0017	0.0814	0.0833	0.0136	0.2254	0.9464	-0.0080
1.15	-0.0017	0.0822	0.0818	0.0135	0.3986	0.9492	-0.0011	0.0803	0.0806	0.0129	0.4200	0.9492	-0.0214
1.20	0.0006	0.0812	0.0805	0.0131	0.6154	0.9522	0.0017	0.0792	0.0785	0.0124	0.6420	0.9522	-0.0266
1.30	0.0000	0.0795	0.0783	0.0125	0.9084	0.9546	0.0032	0.0775	0.0763	0.0118	0.9268	0.9546	-0.0184
1.50	-0.0020	0.0768	0.0779	0.0120	0.9986	0.9462	0.0027	0.0746	0.0753	0.0112	0.9996	0.9462	-0.0010
2.00	0.0009	0.0732	0.0752	0.0110	1.0000	0.9436	0.0104	0.0705	0.0722	0.0103	1.0000	0.9436	0.0000
Random Censoring, 10% Cumulative Disease Incidence (if there was no censoring)													
1.00	-0.0001	0.0710	0.0722	0.0103	0.0558	0.9532	-0.0005	0.0676	0.0683	0.0092	0.0468	0.9532	0.0090
1.05	-0.0016	0.0701	0.0690	0.0097	0.1018	0.9536	-0.0009	0.0668	0.0664	0.0089	0.1150	0.9536	-0.0132
1.10	-0.0011	0.0694	0.0680	0.0094	0.2714	0.9506	-0.0004	0.0660	0.0643	0.0085	0.2964	0.9506	-0.0250
1.15	-0.0021	0.0688	0.0703	0.0097	0.5156	0.9438	-0.0006	0.0653	0.0670	0.0088	0.5660	0.9438	-0.0504
1.20	-0.0004	0.0681	0.0676	0.0092	0.7638	0.9536	0.0026	0.0647	0.0644	0.0083	0.8066	0.9536	-0.0428
1.30	0.0007	0.0671	0.0675	0.0091	0.9730	0.9476	0.0044	0.0636	0.0637	0.0081	0.9860	0.9476	-0.0130
1.50	0.0012	0.0656	0.0667	0.0088	1.0000	0.9466	0.0081	0.0618	0.0631	0.0079	1.0000	0.9466	0.0000
2.00	0.0000	0.0637	0.0640	0.0082	1.0000	0.9438	0.0137	0.0592	0.0590	0.0072	1.0000	0.9438	0.0000
Random Censoring, 15% Cumulative Disease Incidence (if there was no censoring)													
1.00	-0.0013	0.0653	0.0648	0.0085	0.0446	0.9542	-0.0024	0.0609	0.0603	0.0074	0.0458	0.9542	-0.0012
1.05	0.0004	0.0647	0.0648	0.0084	0.1214	0.9498	0.0007	0.0603	0.0601	0.0072	0.1322	0.9498	-0.0108
1.10	0.0018	0.0641	0.0636	0.0082	0.3278	0.9534	0.0025	0.0597	0.0590	0.0071	0.3646	0.9534	-0.0368
1.15	0.0003	0.0636	0.0640	0.0081	0.5976	0.9418	0.0029	0.0592	0.0598	0.0071	0.6796	0.9418	-0.0820
1.20	0.0002	0.0632	0.0624	0.0079	0.8260	0.9540	0.0033	0.0588	0.0580	0.0068	0.8928	0.9540	-0.0668
1.30	0.0019	0.0625	0.0626	0.0078	0.9888	0.9470	0.0064	0.0579	0.0585	0.0068	0.9960	0.9470	-0.0072
1.50	0.0001	0.0614	0.0629	0.0077	1.0000	0.9424	0.0080	0.0567	0.0583	0.0067	1.0000	0.9424	0.0000
2.00	0.0000	0.0605	0.0608	0.0074	1.0000	0.9366	0.0168	0.0550	0.0554	0.0064	1.0000	0.9366	0.0000

HR, hazard ratio; OR, odds ratio; RAF, risk allele frequency; Diff in Power, difference in power (Cox - logistic); SD, standard deviation; SE, standard error of the logarithm of hazard or odds ratio; MSE, mean squared error (MSE=$Bias^2$+SE^2). Bias refers to either the estimated log(HR) or log(OR) minus the underlying log(HR). It is important to note that HRs and ORs are different measures of comparison and as such ORs are not ?biased? if different from the underlying HR. Coverage refers to the proportion of 95% confidence intervals that contain the underlying association. The Cox model was Prentice weighted and robust SEs were used to account for the sampling process.

Table S7: Computational time for 10,000 SNPs

Model	Cumulative Disease Incidence	Cohort Study (seconds) [N=10,000]	Case-cohort Study (seconds) [θ=15%; N (Original Cohort)=40,000]
Logistic	5%	119	84
Regression	10%	119	98
	15%	119	118
Cox	5%	2238	16180
Regression	10%	2180	30040
	15%	2187	41604

N, number of individuals; θ, sampling fraction. The Cox model is Prentice weighted and robust standard errors were used in the case-cohort study setting.

Table S8: Results of 25 SNPs previously reported to be associated with CHD in EPIC-CVD and findings from the CARDIoGRAM, C4D, and CARDIoGRAMplusC4D consortia.

		EPIC-CVD								CARDIO		C4D		CARDIO+C4D	
		Subcohort				Case-cohort									
		Cox		Logistic		Cox		Logistic							
rsID	Build 37 chr:pos	β	p	β	p	β	p	β	p	β	p	β	p	β	p
rs11206510	chr1:g.55496039T>C	0.058	0.509	0.052	0.572	0.069	0.054	0.073	0.021	0.080	4.8E-4	0.051	0.052	0.055	1.9E-5
rs4299376	chr2:g.44072576T>G	-0.020	0.785	-0.011	0.885	-0.058	0.053	-0.043	0.098	-0.053	9.5E-4	-0.079	1.8E-5	-0.054	2.9E-7
rs2252641	chr2:g.145801461T>C	-0.130	0.057	-0.138	0.054	-0.019	0.508	-0.012	0.639	-0.060	1.4E-5	-0.033	0.051	-0.048	2.6E-8
rs2306374	chr3:g.138119952T>C	-0.116	0.199	-0.125	0.189	0.008	0.843	-0.008	0.821	-0.108	3.3E-8	-0.080	1.5E-3	-0.073	2.4E-9
rs273909	chr5:g.131667353A>G	0.012	0.909	-0.001	0.996	-0.049	0.260	-0.065	0.083	-0.065	3.2E-3	-0.037	0.222	-0.077	5.9E-9
rs2706399	chr5:g.131867702A>G	-0.040	0.552	-0.057	0.428	0.028	0.319	-0.015	0.556	-0.045	5.7E-3	-0.025	0.131	NA	NA
rs9349379	chr6:g.12903957A>G	-0.127	0.066	-0.133	0.069	-0.093	1.2E-3	-0.123	9.5E-7	-0.093	1.5E-7	-0.159	6.5E-21	NA	NA
rs2048327	chr6:g.160863532T>C	0.042	0.553	0.042	0.578	-0.083	5.2E-3	-0.092	3.5E-4	-0.072	4.5E-7	-0.066	2.3E-4	-0.060	4.3E-11
rs2023938	chr7:g.19036775T>C	0.000	1.000	0.001	0.992	-0.018	0.691	-0.043	0.286	-0.079	6.8E-4	-0.015	0.580	-0.073	2.7E-7
rs10953541	chr7:g.107244545C>T	0.061	0.450	0.065	0.438	-0.009	0.792	0.015	0.607	0.033	0.042	0.096	6.0E-6	NA	NA
rs264	chr8:g.19813180G>A	0.140	0.151	0.141	0.168	0.079	0.039	0.076	0.029	0.102	3.0E-7	0.065	4.7E-3	0.071	1.3E-8
rs10808546	chr8:g.126495818C>T	0.033	0.632	0.034	0.642	0.042	0.142	0.036	0.149	0.054	1.6E-4	0.046	0.011	0.048	8.9E-8
rs3217992	chr9:g.22003223C>T	-0.034	0.633	-0.036	0.629	-0.114	8.3E-5	-0.097	1.2E-4	-0.161	1.6E-27	-0.139	3.6E-16	-0.145	3.0E-57
rs2047009	chr10:g.44539913G>T	0.104	0.130	0.106	0.140	0.055	0.049	0.072	3.4E-3	0.061	1.4E-5	0.066	1.0E-4	0.053	7.0E-10
rs1746048	chr10:g.44775824C>T	0.101	0.329	0.110	0.309	0.060	0.146	0.062	0.082	0.083	5.8E-5	0.057	5.7E-3	0.069	6.5E-9
rs11203042	chr10:g.90989109C>T	0.034	0.618	0.037	0.610	0.014	0.612	0.005	0.829	-0.056	7.8E-5	-0.032	0.056	-0.039	8.3E-6
rs1412444	chr10:g.91002927C>T	-0.118	0.097	-0.128	0.086	-0.001	0.962	-0.041	0.111	-0.062	9.1E-4	-0.080	2.8E-6	NA	NA
rs974819	chr11:g.103660567C>T	-0.123	0.091	-0.132	0.086	-0.082	7.3E-3	-0.08	3.1E-3	-0.050	1.2E-3	-0.081	5.7E-6	-0.065	2.0E-11
rs964184	chr11:g.116648917C>G	0.126	0.221	0.128	0.236	-0.047	0.241	-0.063	0.065	-0.126	8.0E-10	-0.057	0.012	NA	NA
rs3184504	chr12:g.111884608C>T	0.049	0.472	0.054	0.447	-0.003	0.919	-0.014	0.560	-0.070	6.4E-6	-0.050	0.056	-0.068	1.8E-11
rs9319428	chr13:g.28973621G>A	-0.158	0.027	-0.165	0.028	-0.050	0.098	-0.057	0.030	-0.062	7.9E-5	-0.047	0.011	-0.055	4.2E-9
rs4380028	chr15:g.79111093C>T	0.082	0.241	0.088	0.229	0.086	2.7E-3	0.065	9.6E-3	0.054	1.7E-4	0.067	1.5E-4	0.057	1.6E-7
rs17514846	chr15:g.91416550C>A	-0.115	0.091	-0.112	0.119	-0.037	0.187	-0.068	6.0E-3	-0.069	2.4E-5	-0.063	2.2E-4	-0.058	2.0E-10
rs6511720	chr19:g.11202306G>T	-0.067	0.525	-0.063	0.568	0.062	0.156	0.067	0.089	0.125	1.6E-4	0.108	1.6E-4	NA	NA
rs2075650	chr19:g.45395619A>G	-0.093	0.347	-0.099	0.340	-0.120	3.1E-3	-0.133	2.4E-4	-0.038	0.196	-0.105	3.0E-5	NA	NA

Build 37 chr:pos, dbSNP (hg19) chromosome-position (risk allele>other allele); β, is the logarithm of the association measure (either odds ratio or hazard ratio); NA, not available. CARDIO C4D and CARDIO+C4D refer to CARDIoGRAM,[7] C4D[8] and CARDIoGRAMplusC4D[9] consortia, respectively. Cox and logistic refer to the model used for the analysis in EPIC-CVD. In the full In the full case-cohort, the Cox model was Prentice weighted and robust standard errors were used to account for the sampling process. Logistic regression was used as the analysis model in each study of the three consortia, regardless of study design. Alleles are aligned to the + strand.

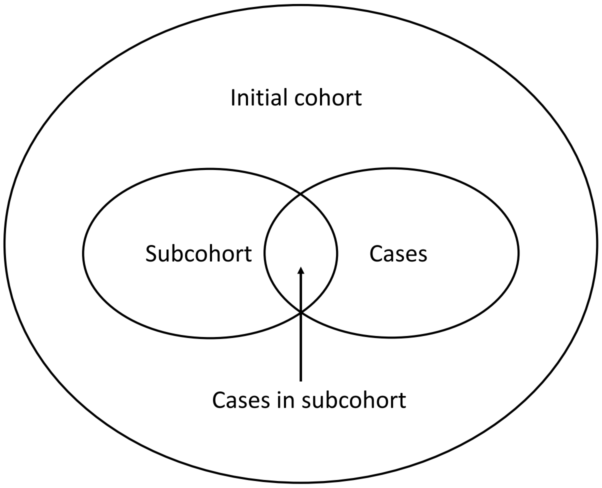

Figure S1 Case-cohort study design.

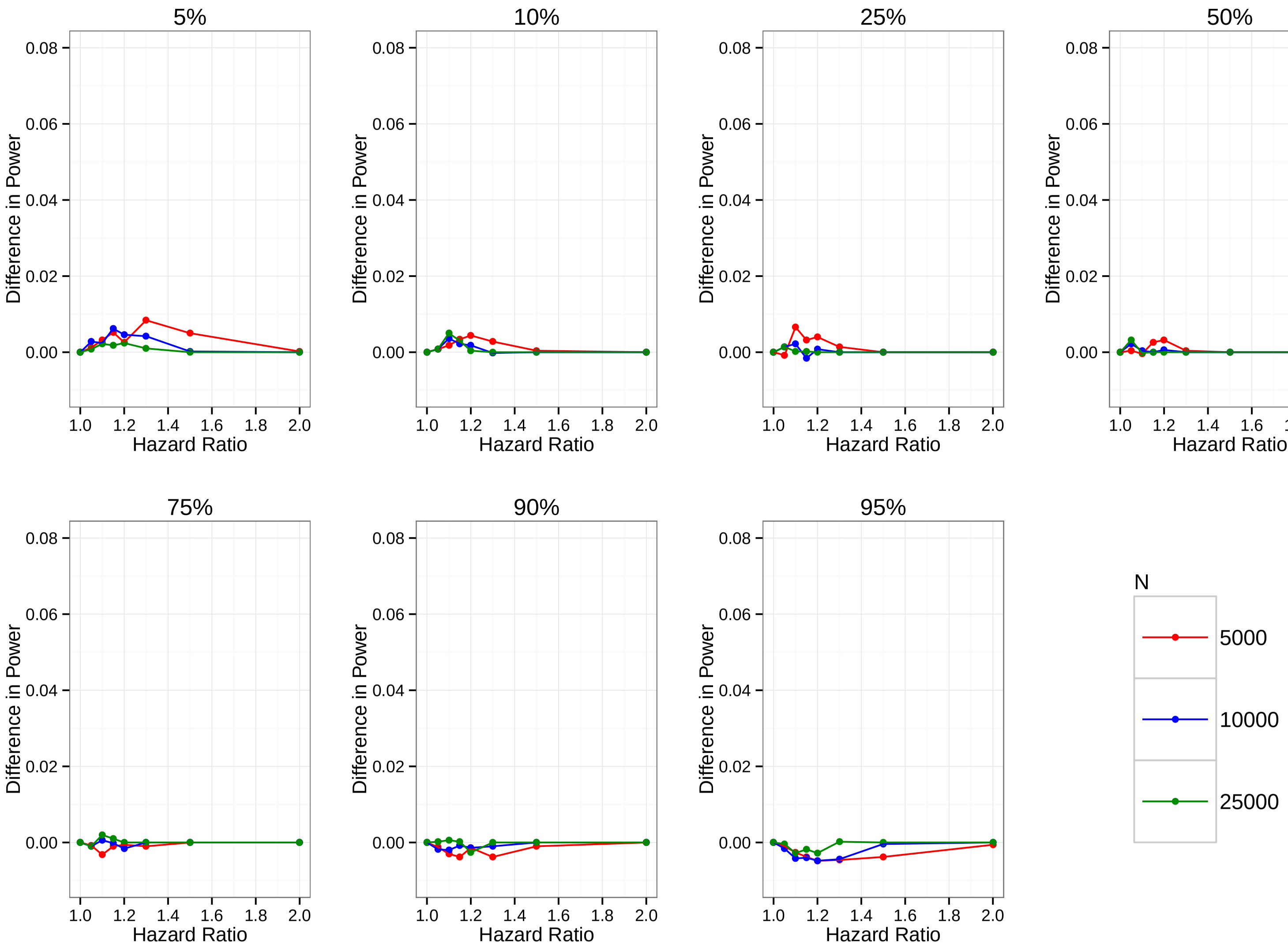

Figure S2 Difference in power between the Cox and logistic regression models for SNPs with a range of risk allele frequencies for the cumulative disease incidence of 10%, complete follow-up model and the cohort study design. The red, blue and green lines represent the sample sizes 5000, 10000 and 25000, respectively. 5%, 10%, 25%, 50%, 75%, 90% and 95% are the risk allele frequencies.

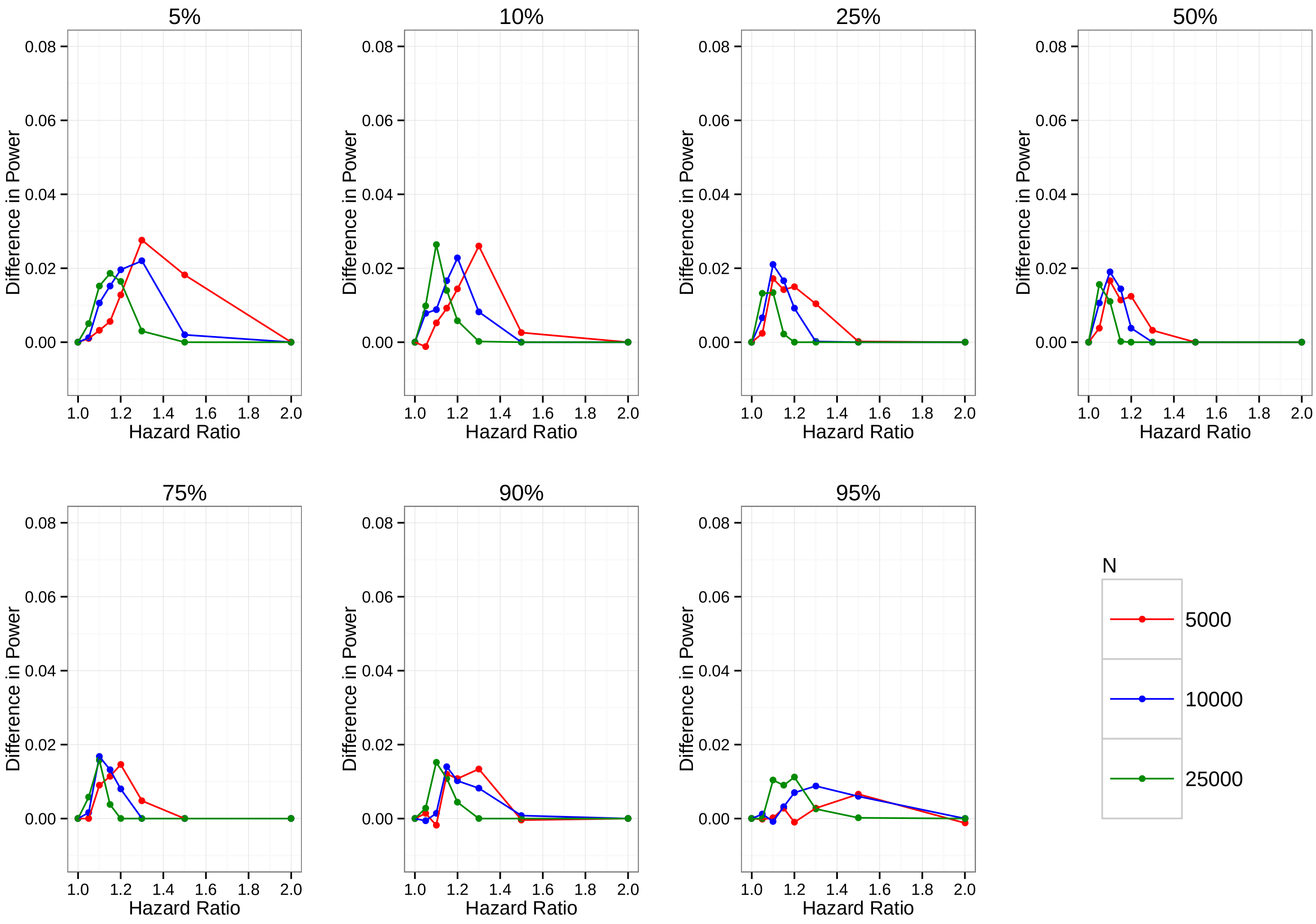

Figure S3 Difference in power between the Cox and logistic regression models for SNPs with a range of risk allele frequencies for the cumulative disease incidence of 10%, survey follow-up model and the cohort study design. The red, blue and green lines represent the sample sizes 5000, 10000 and 25000, respectively. 5%, 10%, 25%, 50%, 75%, 90% and 95% are the risk allele frequencies.

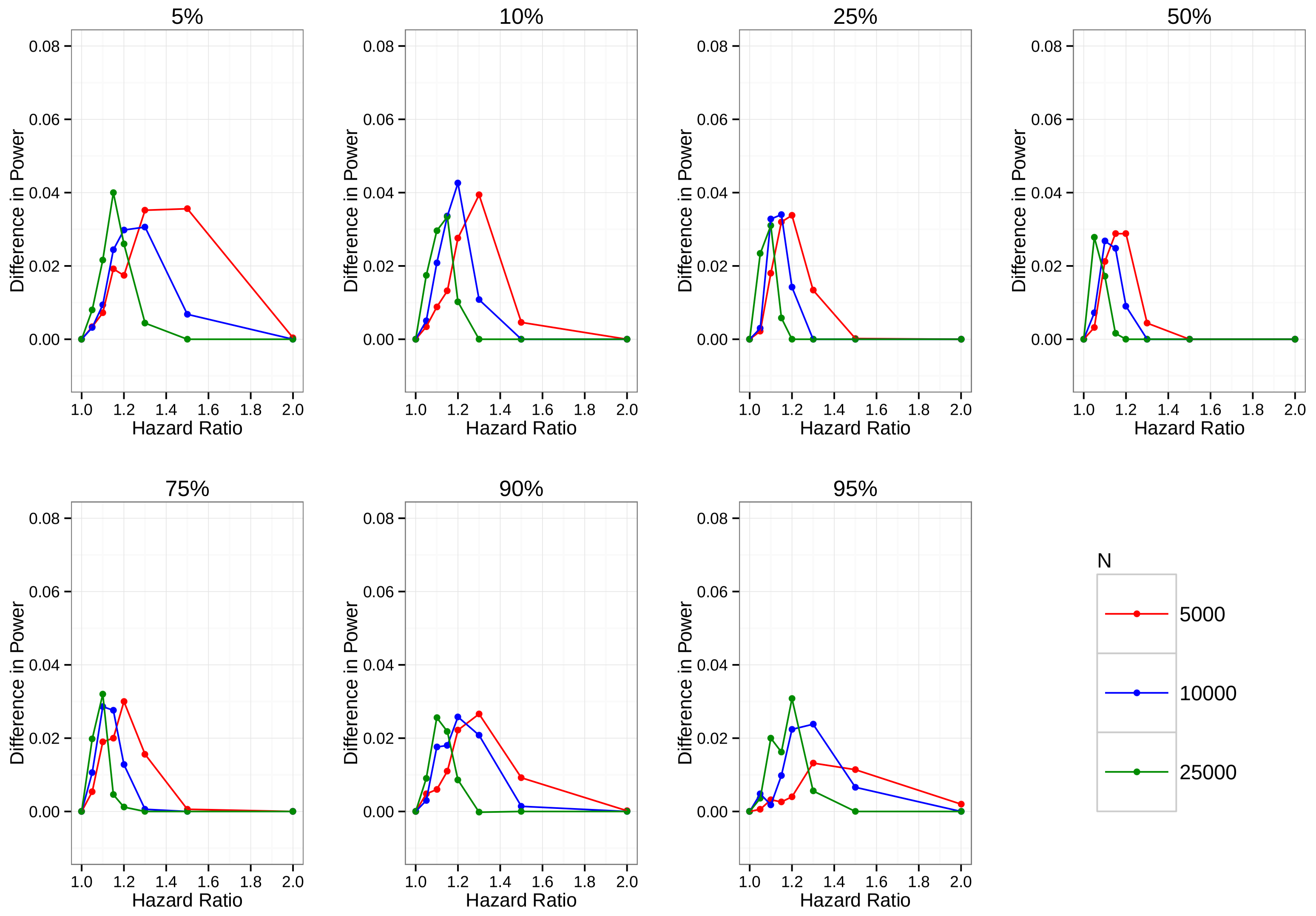

Figure S4 Difference in power between the Cox and logistic regression models for SNPs with a range of risk allele frequencies for the cumulative disease incidence of 10%, random follow-up model and the cohort study design. The red, blue and green lines represent the sample sizes 5000, 10000 and 25000, respectively. 5%, 10%, 25%, 50%, 75%, 90% and 95% are the risk allele frequencies.

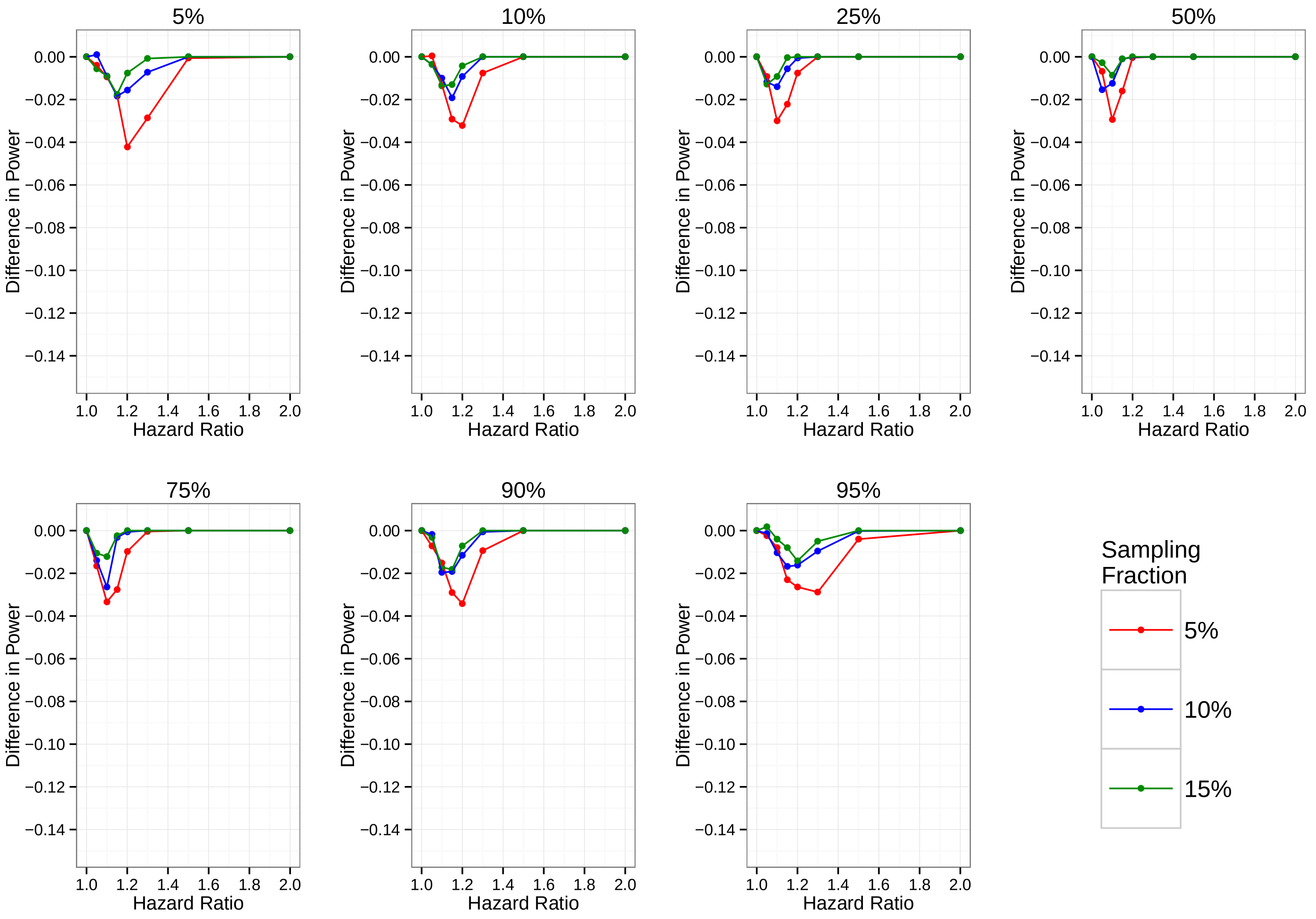

Figure S5 Difference in power between the Cox and logistic regression models for SNPs with a range of risk allele frequencies for the cumulative disease incidence of 10%, complete follow-up model and the case-cohort study design. The red, blue and green lines represent the sampling fractions of 5%, 10% and 15%, respectively. 5%, 10%, 25%, 50%, 75%, 90% and 95% are the risk allele frequencies.

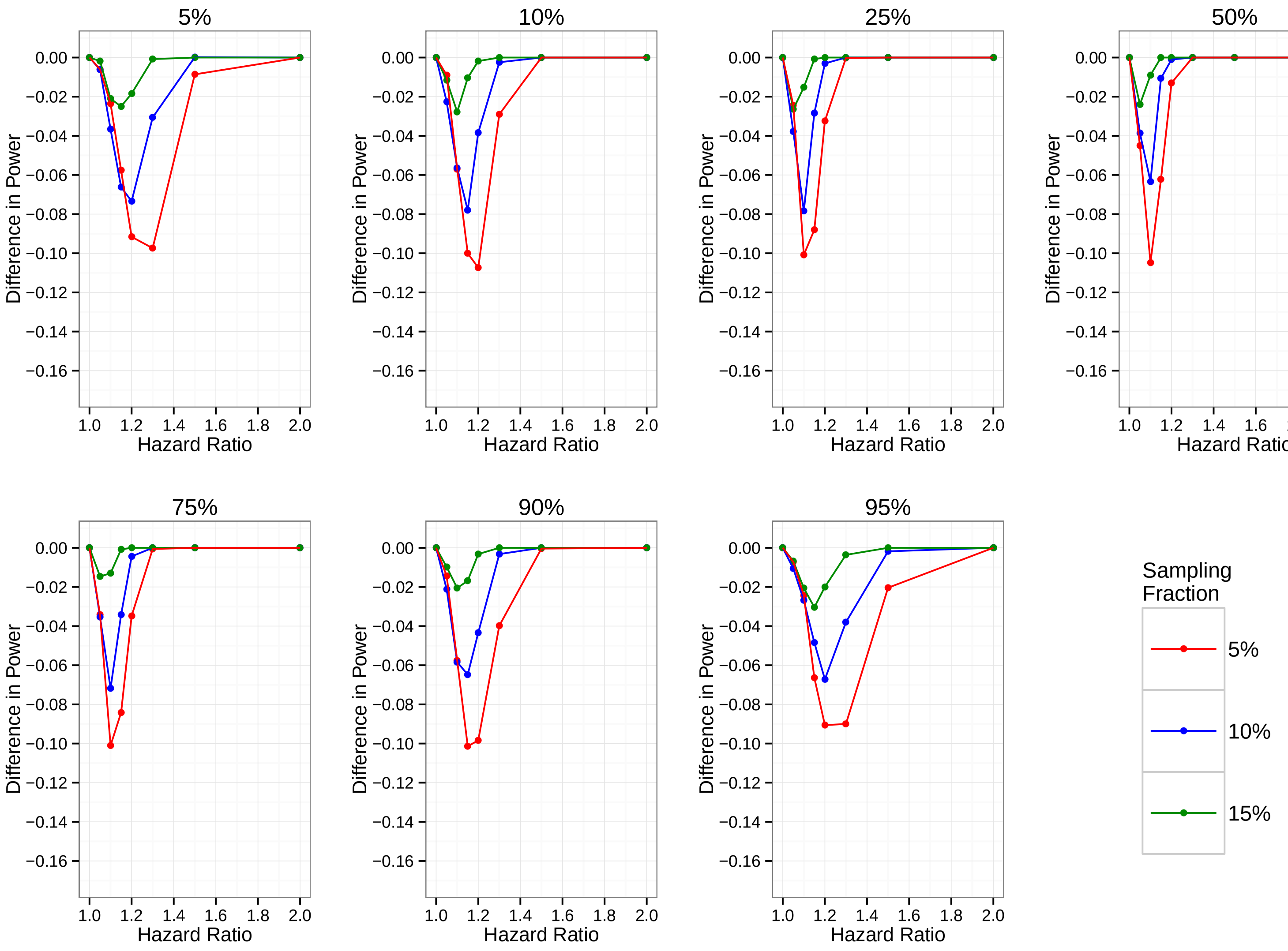

Figure S6 Difference in power between the Cox and logistic regression models for SNPs with a range of risk allele frequencies for the cumulative disease incidence of 10%, survey follow-up model and the case-cohort study design. The red, blue and green lines represent the sampling fractions of 5%, 10% and 15%, respectively. 5%, 10%, 25%, 50%, 75%, 90% and 95% are the risk allele frequencies.

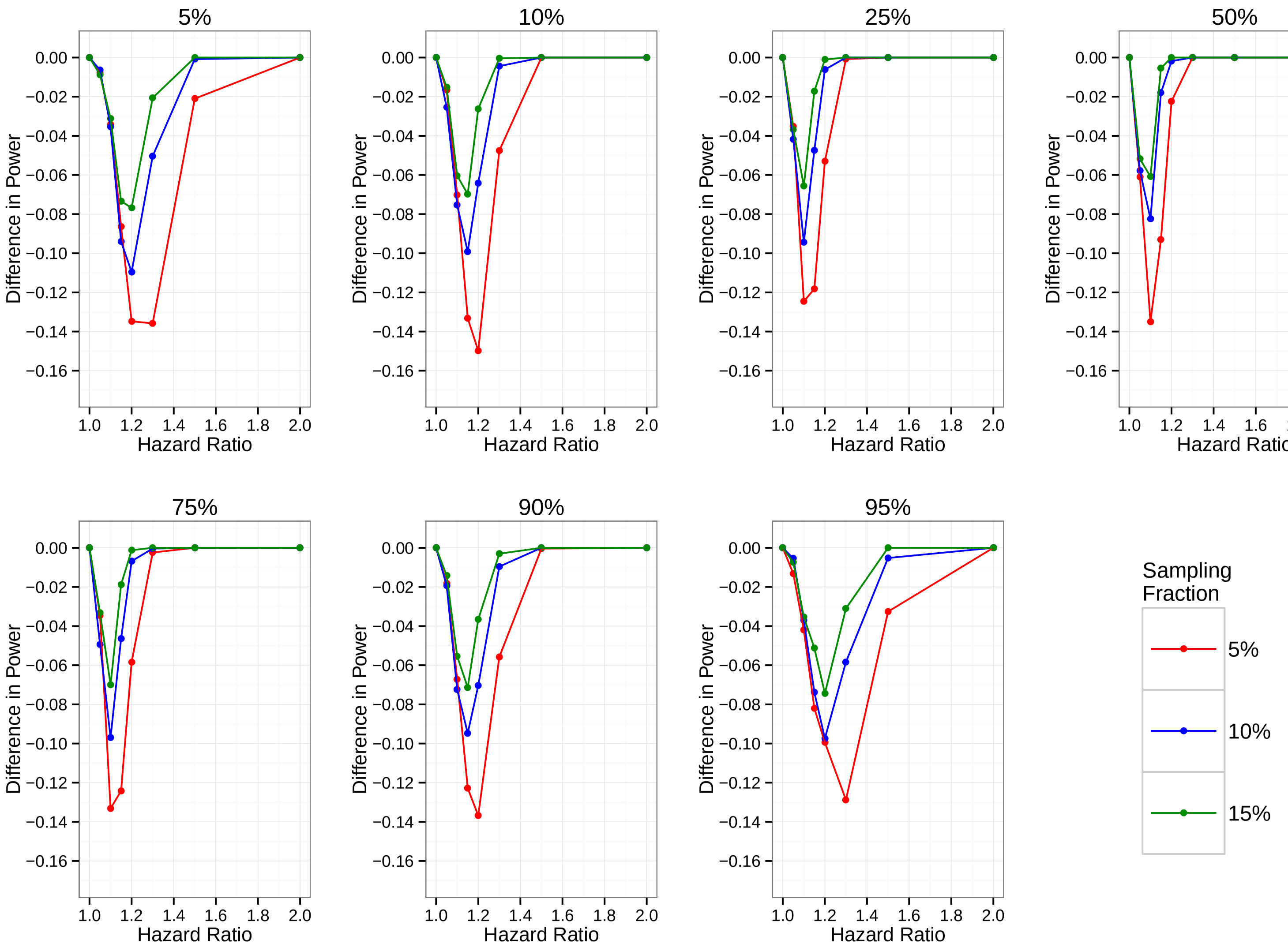

Figure S7 Difference in power between the Cox and logistic regression models for SNPs with a range of risk allele frequencies for the cumulative disease incidence of 10%, random follow-up model and the case-cohort study design. The red, blue and green lines represent the sample sizes 5%, 10% and 15%, respectively. 5%, 10%, 25%, 50%, 75%, 90% and 95% are the risk allele frequencies.

References

1. Cox DR. The regression analysis of binary sequences. *J R Statist Soc B*. 1958;20:215–242.

2. Walker SH, Duncan DB. Estimation of the probability of an event as a function of several independent variables. *Biometrika*. 1967;54(1):167–179.

3. Prentice RL. A case-cohort design for epidemiologic cohort studies and disease prevention trials. *Biometrika*. 1986;73(1):1–11.

4. Cox DR. Regression models and life tables (with discussion). *J R Statist Soc*. 1972;34:187–220.

5. Onland-Moret NC, van der A DL, van der Schouw YT, Buschers W, Elias SG, van Gils CH, et al. Analysis of case-cohort data: a comparison of different methods. *J Clin Epidemiol*. 2007;60(4):350–355.

6. Barlow WE, Ichikawa L, Rosner D, Izumi S. Analysis of case-cohort designs. *J Clin Epidemiol*. 1999;52(12):1165–1172.

7. Schunkert H, König IR, Kathiresan S, Reilly MP, Assimes TL, Holm H, et al. Large-scale association analysis identifies 13 new susceptibility loci for coronary artery disease. *Nat Genet*. 2011;43(4):333–338.

8. Coronary Artery Disease (C4D) Genetics Consortium, et al. A genome-wide association study in Europeans and South Asians identifies five new loci for coronary artery disease. *Nat Genet*. 2011;43(4):339–344.

9. Deloukas P, Kanoni S, Willenborg C, Farrall M, Assimes TL, Thompson JR, et al. Large-scale association analysis identifies new risk loci for coronary artery disease. *Nat Genet*. 2013;45(1):25–33.
